# Ethnobotanical study on medicinal plants used by Jinuo people in Yunnan, China

**DOI:** 10.1186/s13002-026-00927-z

**Published:** 2026-07-07

**Authors:** Qing Huang, Wei Liu, Youwen Li, Jiaqi Zhang, Xiran Wu, Junxi Wang, Zhenghong Yu, Qingsong Zhao, Liying Yang, Lun Yin, Weiwei Li

**Affiliations:** 1 Institute of National Security, Minzu University of China, Beijing, 100081 China; 2 School of Management, Minzu University of China, Beijing, 100081 China; 3 College of Marxism, Southwest Forestry University, Kunming, 650233 China; 4 Biodiversity Museum of Hainan Normal University, Haikou, 571158 China; 5 Ministry of Education Key Laboratory for Ecology of Tropical Islands, Key Laboratory of Tropical Animal and Plant Ecology of Hainan Province, College of Life Sciences, Hainan Normal University, Haikou, 571158 China; 6 College of Soil and Water Conservation, Southwest Forestry University, Kunming, 650233 China; 7 Yunnan International Joint Center of Urban Biodiversity, Yuxi, 652799 Yunnan China; 8 Southwest Ecological Civilization Research Center, National Forestry and Grassland Administration, Kunming, 650233 China; 9 Yunnan Key Laboratory of Biodiversity Information, Kunming Institute of Zoology, The Chinese Academy of Sciences, Kunming, 650201 China

**Keywords:** Jinuo people, Ethnobotany, Medicinal plants, Traditional medicine, Traditional knowledge

## Abstract

**Background:**

Traditional medicine is rooted in knowledge systems developed over time across diverse cultures, serving as an important source of healthcare practices and therapeutic methods. Ethnic medicine, in particular, reflects rich experience in the use of medicinal plants and has significant practical value. The medicinal plants of the Jinuo people and their associated traditional knowledge are an important part of China’s ethnomedical system, yet systematic studies are still limited. This study therefore conducts an ethnobotanical survey in Jinuo communities to document and analyze their use of medicinal plants and associated traditional knowledge.

**Methods:**

The fieldwork was conducted from October 2021 to June 2025 in the Jinuo Ethnic Township of Jinuo Mountain, covering 12 villages. A total of 213 local residents were surveyed, including 126 men and 87 women. Thirteen traditional Jinuo healers were selected as key informants through purposive sampling. In addition, semi-structured interviews and group discussions were conducted with 200 additional informants from the Jinuo community. Finally, the collected ethnobotanical data were analyzed using relative frequency of citation (RFC).

**Results:**

A total of 226 medicinal plant species were recorded in the study area, belonging to 197 genera and 90 families, with Fabaceae, Asteraceae, and Lamiaceae as the dominant families. Herbaceous plants constituted the majority of medicinal species. Thirteen plant parts were used for medicinal preparation, among which roots were the most frequently used, followed by whole plants. The results showed that these medicinal plants were used to treat 94 human diseases, with the primary preparation methods being decoction for oral administration and fresh application by pounding. To assess the relative importance of medicinal plants locally, RFC values were calculated for each species. Among them, *Plantago asiatica* (0.87), *Stephania epigaea* (0.86), *Tacca chantrieri* (0.85), *Gynostemma pentaphyllum* (0.85), and *Phyllanthus urinaria* (0.84) showed relatively high RFC values. In addition, the survey recorded 40 common medicinal and edible plants in the local community.

**Conclusion:**

This study shows that the Jinuo people possess rich medicinal plant resources, which play an important role in treating common ailments and maintaining daily health. However, the aging of knowledge holders, the decline in the number of traditional Jinuo healers, insufficient transmission to younger generations, and increasing human disturbances are leading to the continuous loss of medicinal plant resources and associated traditional knowledge. Therefore, it is urgent to strengthen the conservation of medicinal plant resources, as well as the documentation and transmission of traditional knowledge systems, in order to promote the sustainable use of Jinuo traditional medicine and provide references for the protection of ethnomedicine and the development of biological resources.

## Introduction

The World Health Organization (WHO) describes traditional medicine as an umbrella term encompassing systems such as traditional Chinese medicine, Indian medicine, and Arabic medicine, as well as various forms of folk therapy. It defines traditional medicine as the sum total of the knowledge, skills and practices based on the theories, beliefs and experiences indigenous to different cultures, whether explicable or not, used in the maintenance of health, as well as in the prevention, diagnosis, improvement or treatment of physical and mental illnesse [1, 2]. In China, traditional medicine consists of three main components: traditional Chinese medicine, folk medicine, and ethnic medicine [3]. Traditional Chinese medicine, rooted in Han culture, was the mainstream medical system in ancient Chinese society. After a long course of development, it continues to occupy a central position in both academic research and social practice, and is widely recognized as the principal representative of Chinese traditional medicine [4]. Folk medicine refers to folk healing experience, health-preserving customs, empirically tested remedies, and simple therapeutic techniques that lack both a formal medical theoretical system and a distinct ethnic cultural background. It is not necessarily guided by the theories of traditional Chinese medicine, nor can it easily be classified as any particular form of ethnic medicine [5]. Ethnic medicine refers to the traditional medical practices of China’s ethnic minority groups and, together with traditional Chinese medicine, constitutes an important component of Chinese traditional medicine. China is home to 55 ethnic minority groups, each of which has accumulated rich knowledge and experience in disease prevention and treatment through long-term productive and everyday practices, thereby forming distinctive ethnic medical systems [6].

From the perspective of scholarly development, ethnic medicine in China can generally be classified into three categories. The first comprises ethnic medical traditions with relatively well-established theoretical systems and clinical practices, supported by systematic historical medical literature and having developed into relatively mature disciplinary systems, such as Tibetan, Mongolian, Uyghur, and Dai medicine. The second comprises ethnic medical traditions whose theoretical systems have not yet been fully systematized but which possess a certain documentary basis and rich clinical experience, such as Zhuang, Miao, Yi, and She medicine. The third includes ethnic medical traditions that are mainly transmitted orally within communities across generations and lack systematic written records, as well as medical techniques and medicinal knowledge that have gradually developed distinctive ethnic characteristics through the incorporation of traditional Chinese medicine theory, such as Shui, Jinuo, and Bai medicine [7, 8]. 

Ethnic medical culture represents the cumulative wisdom developed by China’s ethnic minority groups through their long struggle against disease, and it has played an important role in safeguarding the health, reproduction, and social development of these communities. In the course of gathering plants, hunting animals, and consuming them as food, ethnic minority groups in China occasionally discovered that certain plants and animals could relieve poisoning or alleviate other adverse symptoms, and thus gradually came to recognize their potential medicinal value. Through repeated experimentation and the accumulation of experience, they progressively developed the ability to identify and purposefully utilize animal and plant resources, thereby acquiring medical knowledge for the relief of illness and pain and promoting the formation and development of ethnic medicine [9]. Regions inhabited by ethnic minority groups are usually rich in plant resources. Plants serve not only as an important source of food for local residents, but also as a major source of medicines for the prevention and treatment of disease [10]. The human use of medicinal plants has a history of hundreds or even thousands of years. Medicinal plants are widely regarded as an effective means of preventing and treating a variety of health problems, and many modern medicines are derived directly or indirectly from traditional herbal remedies. These remedies are still widely used today for health maintenance and as complementary therapeutic resources [11]. 

Medicinal plant resources constitute an essential material foundation for human health. Far from declining or disappearing over the course of history, medicinal plants have become increasingly important in human life over thousands of years [12]. According to WHO statistics, approximately 80% of the global population rely on traditional medicine or complementary and alternative therapies, and 50% have used traditional medicinal products [13, 14]. At the same time, traditional medicines constitute a major source for new drug discovery, and drug-screening approaches guided by traditional medical knowledge or the natural habitats of medicinal resources are markedly more effective than random discovery models [15–17]. However, the development of traditional medicine depends heavily on the sustained supply of medicinal plant resources [18]. Therefore, promoting the comprehensive development of traditional medicine requires the sustainable management of medicinal plant resources, while the traditional knowledge closely associated with these resources is equally important [19]. Traditional knowledge of medicinal plants among the ethnic group is not only an important part of ethnic cultural heritage, but also of potential value for safeguarding biological resource security and national security. 

Xishuangbanna Dai Autonomous Prefecture is rich in medicinal plant resources. A total of 1,724 species of Chinese medicinal plants have been recorded in the region, more than 500 of which have been identified [20]. It is therefore widely known as a “Plant Kingdom” and a “Biological Gene Bank.” As a typical frontier multi-ethnic region, Xishuangbanna is home to 13 long-established ethnic groups. Over the course of their long struggle against disease, these communities have accumulated rich medical knowledge and developed a variety of traditional therapeutic practices with distinctive ethnic and local characteristics. At present, the regional medical system is dominated by Dai medicine, with Jinuo, Bulang, Hani, and other forms of ethnic medicine playing supplementary roles [21]. The Jinuo, the last ethnic minority officially recognized by the State Council of China, are mainly concentrated in Jinuo Mountain Jinuo Ethnic Township, Jinghong, Xishuangbanna. The region is characterized by a hot and humid climate, fertile soil, and abundant plant resources [22]. Historically, Jinuo Mountain was plagued by miasma and poor access to medicine and healthcare. In the course of long-term agricultural practice and repeated struggles against disease, the Jinuo gradually accumulated experience in the use of herbal medicine, thereby giving rise to Jinuo medical culture [23]. According to the classification outlined above, Jinuo medicine belongs to the third category of ethnic medicine. 

Jinuo medicine is a traditional form of ethnic medicine characterized by a strong practical and experiential orientation. Through the slaughter and anatomical observation of various animals, and by comparing animal structures with those of the human body, the Jinuo developed a relatively clear understanding of human anatomy [23]. At the same time, they accumulated extensive knowledge of herbal medicines through everyday production and daily life. For example, the tender leaves of *Psidium guajava.* are used to treat diarrhea [21]. However, because the Jinuo historically had no written language, Jinuo medicine has been transmitted mainly through apprenticeship and oral instruction. Under the impact of external cultures, Jinuo traditional knowledge, especially knowledge related to medicinal plants, is now disappearing. Large numbers of young Jinuo people leave their communities for schooling or employment, which reduces their opportunities to learn herbal knowledge from elders. In addition, the development of modern medicine has made people more inclined to seek treatment at clinics [24]. Although many ethnobotanical surveys have been conducted in ethnic minority regions of China, few have focused on medicinal plants used by the Jinuo people. A systematic investigation of Jinuo medicinal plants and associated traditional knowledge is urgently needed. Therefore, this study aims to: (1) systematically document Jinuo medicinal plants and associated traditional knowledge to provide baseline data for resource conservation and future research; (2) identify key medicinal plants in local healthcare practices using RFC values and evaluate the research and application status of ethnobotanically important species; (3) assess major threats to medicinal plant resources and traditional knowledge transmission, and propose conservation strategies. 

This study proposes three hypotheses: (i) medicinal plants with higher RFC values are more important in local healthcare practices and may have greater potential for phytochemical, pharmacological, or resource-development studies; (ii) the use of medicinal food plants by the Jinuo people reflects the interaction between local dietary culture, ecological adaptation, and household healthcare practices; (iii) Jinuo medicinal plant knowledge shares similarities with that of neighboring or ecologically similar regions, but also differs due to ethnic culture, disease perception, and knowledge transmission.

## Methods

### Study area

Xishuangbanna Dai Autonomous Prefecture (21°08′–22°36′ N, 99°56′–101°50′ E) is located in the southernmost part of Yunnan Province. It comprises Jinghong City, Menghai County, and Mengla County. The region has long been inhabited by multiple ethnic groups, including the Dai, Hani, Blang, and Jinuo peoples. The Jinuo people are the last officially recognized single ethnic group in China and are also an indigenous ethnic group unique to Yunnan. They are mainly distributed in Jinuo Mountain Jinuo Ethnic Township and its surrounding areas in Jinghong City, with a small number living in Menglun, Xiangming, and other areas of Mengla County [25]. The Jinuo people have their own ethnic language. The Jinuo language belongs to the Yi branch of the Tibeto-Burman language family within the Sino-Tibetan language family. The Jinuo people do not have their own written script. In the past, they often used carved wood and bamboo to keep counts and record events [26]. According to the China Statistical Yearbook 2021, the Jinuo population in China was 26,025 [27].

**Fig. 1:**
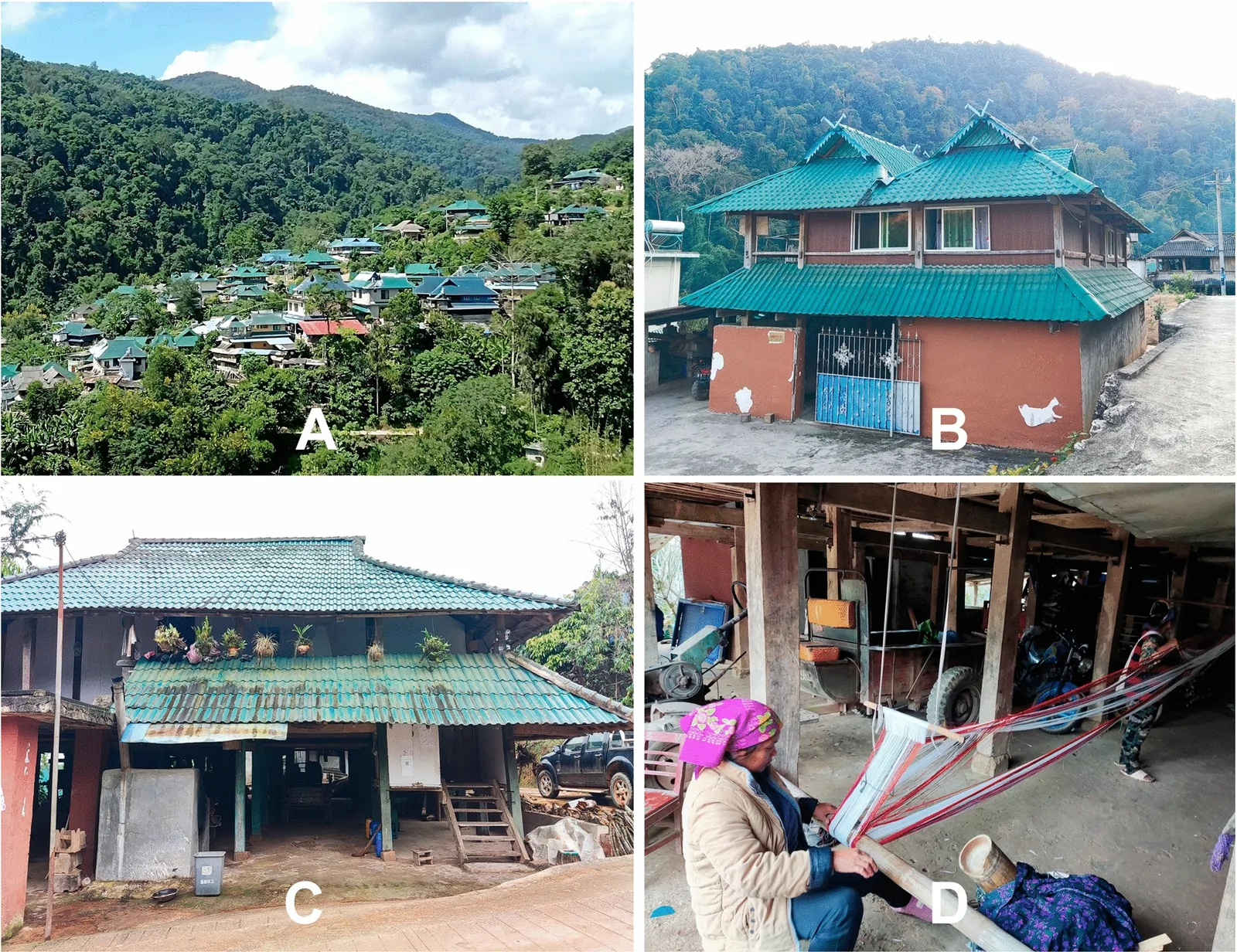
**A** Aerial view of Bakaxiaozhai village, **B** Modernized stilted dwelling of the Jinuo ethnic group, **C** Traditional stilted dwelling of the Jinuo ethnic group, **D** Ground floor space for storage and traditional weaving

The present study focuses on Jinuo Mountain Jinuo Ethnic Township in northeastern Jinghong, approximately 27 km from the city center. It is the place of origin of the Jinuo people and their main settlement area. The township covers 619.2 km^2^, more than 95% of which is mountainous terrain with slopes above 25°, making it a typical mountainous ethnic township [25]. The local ecosystem is relatively well preserved, with a forest coverage rate of 94.01%. Elevation ranges from 550 to 1,691 m. The mean annual temperature is 18–20 °C, annual precipitation is about 1,100 mm, and annual sunshine averages 1,852.14 h. The climate is mild and humid, frost-free throughout the year, and marked by distinct wet and dry seasons, with the rainy season lasting from May to September and peak rainfall occurring in July and August [28]. Owing to the hot and humid climate, traditional Jinuo houses are mainly stilt dwellings. These are usually two-storey structures, with the lower level used for storage or livestock and the upper level for living. This design allows for good ventilation and helps reduce dampness and heat, making it well adapted to the local environment. Villages are typically built along mountain slopes, with houses surrounded by dense forest, forming a traditional settlement landscape with distinctive local characteristics (Fig. 1).

**Fig. 2:**
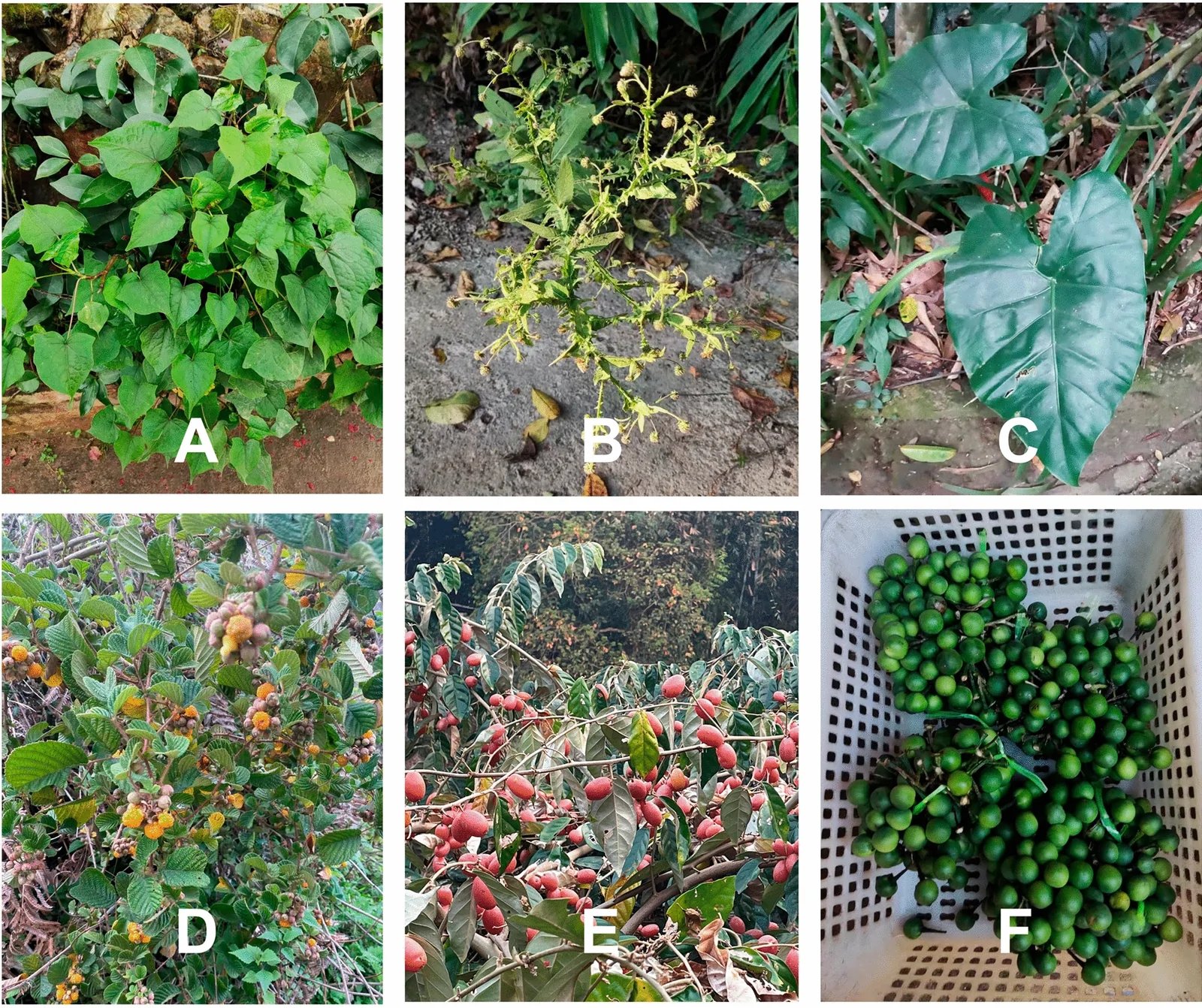
Medicinal plants around local villages in the study area. **A** *Fagopyrum dibotrys* (D. Don) Hara, **B** *Laggera crispata* (Vahl) Hepper & J. R. I. Wood, **C** *Alocasia odora* (Roxb.) K. Koch, **D** *Rubus ellipticus* var. *obcordatus* (Franch.) Focke, **E** *Elaeagnus conferta* Roxb., **F** *Solanum torvum* Sw

The warm and humid climate, together with the complex mountainous terrain, provides favorable conditions for the formation of diverse plant communities. The vegetation of Jinuo Mountain is dominated by tropical montane rainforest and evergreen broad-leaved forest. More than 1,000 forest tree species and over 2,000 economic plant species have been recorded in the area [28], and medicinal plants are widely distributed in the study area (Fig. 2), providing an important material basis for the accumulation and transmission of traditional ethnic medical knowledge. In terms of traditional livelihoods, the Jinuo people have long relied primarily on mountain shifting cultivation, with upland rice and maize as the main crops [29]. Hunting, fishing, and gathering also formed part of their subsistence economy. Among these activities, gathering was especially important in women’s daily work and household life. The collection of wild vegetables, fruits, and fungi not only provided food but also helped sustain local knowledge of plants used for both dietary and medicinal purposes [30]. The complex mountainous environment and rich plant resources of Jinuo Mountain have therefore played an important role in shaping Jinuo residents’ experience in plant identification, collection, processing, and use. Agricultural practice was also closely guided by observations of seasonal and ecological changes. Rather than following a fixed calendar alone, local people traditionally determined the timing of farming activities by observing natural signals such as plant sprouting and flowering, insect calls, and grain ripening. These observations informed key farming practices, from forest clearing and burning to sowing, weeding, and harvesting [31]. Over time, they developed into a body of empirical phenological knowledge closely connected with the local ecological environment.

With social and economic development, paddy fields were gradually opened up and rice cultivation expanded. Tropical cash crops such as rubber and *Amomum villosum* were introduced, while the scale of tea cultivation also continued to increase. These changes contributed to the gradual optimization of the local agricultural structure. The Jinuo people have a long history of tea cultivation and tea use, and Jinuo Mountain is one of the six famous ancient tea mountains of Pu’er tea [32]. Tea is not only an important cash crop but is also deeply embedded in Jinuo people’s daily life and health beliefs. Local people generally believe that tea has health-promoting effects, including clearing internal heat, detoxifying the body, promoting digestion, and relieving greasiness. Through long-term productive and everyday practices, they have developed a distinctive “tea-eating culture,” characterized by tea drinking and the consumption of fresh tea leaves. This culture reflects the traditional use of plant resources as both food and medicine [33].

### Field surveys and ethnobotanical data collection

**Fig. 3:**
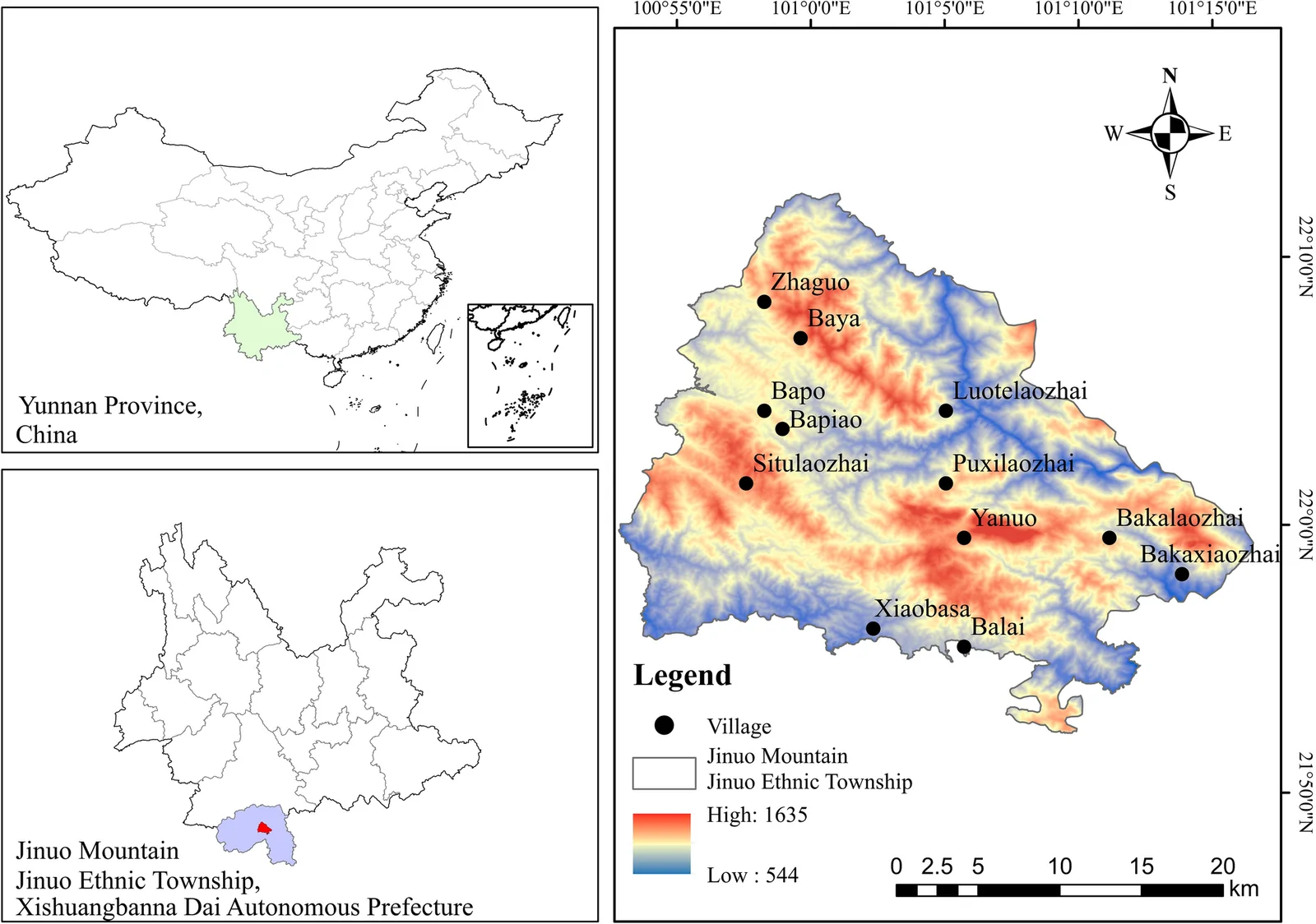
The study area

Through a review of relevant books and literature, we preliminarily compiled an inventory of medicinal plants used by the Jinuo people. Between October 2021 and June 2025, the research team conducted five field investigations in the Jinuo Mountain area of Jinghong City, Xishuangbanna Dai Autonomous Prefecture, to verify and supplement information on local medicinal plants and to collect related ethnobotanical data. The survey covered 12 villages (Fig. 3). Basic information on the surveyed settlements, including settlement name, GPS, altitude, ecology, language, number of households, number of inhabitants, and the number and gender distribution of study participants, is presented in Table 1. A total of 213 local residents were interviewed, including 126 men and 87 women. Their demographic characteristics are summarized in Table 2. Among them, 13 traditional Jinuo healers were selected as key informants through purposive sampling for in-depth interviews. Of these 13 healers, only two were women, and they mainly specialized in the treatment of traumatic injuries and gynecological disorders. Some healers had simple wards in their homes. In their clinical practice, they often combined biomedical diagnostic information with traditional ethnic herbal medicine, which helped improve the clarity of treatment practices and, to some extent, enhanced the credibility of ethnic medicine, thereby contributing to the development of traditional Jinuo medicine. In addition, 200 additional informants were selected from the villages through snowball sampling for interviews. Information such as the respondents’ names, ages, and knowledge of medicinal plants was recorded. Prior to the survey, informed consent was obtained from all participants, and the interview process was recorded and photographed with their permission. All interviewees were local residents between 18 and 80 years of age.

**Table 1:** Basic information on the surveyed settlements

Settlement name	GPS	Altitude	Ecology	Language	Number of households	Number of inhabitants	Number of study participants	
Xiaobasa	21°56′10.92″N, 101°02′31.75″E	720m	Mountainous area in a tropical rainforest region	Youle Jinuo; Mandarin Chinese	53	251	12	Male 7
								Female 5
Bakalaozhai	21°59′11.33″N, 101°10′19.41″E	1135m	Mountainous area in a tropical rainforest region	Youle Jinuo; Mandarin Chinese	79	305	18	Male 10
								Female 8
Bakaxiaozhai	21°58′01.40″N, 101°12′43.26″E	664m	Mountainous area in a tropical rainforest region	Youle Jinuo; Mandarin Chinese	58	210	15	Male 9
								Female 6
Bapo	22°03′23.97″N, 100°59′07.92″E	1062m	Mountainous area in a tropical rainforest region	Youle Jinuo; Mandarin Chinese	109	415	25	Male 13
								Female 12
Baya	22°05′49.00″N, 101°00′07.80″E	1079m	Mountainous area in a tropical rainforest region	Youle Jinuo; Mandarin Chinese	128	410	20	Male 12
								Female 8
Situlaozhai	22°01′01.85″N, 100°58′20.09″E	1245m	Mountainous area in a tropical rainforest region	Youle Jinuo; Mandarin Chinese	128	310	16	Male 10
								Female 6
Yanuo	21°59′21.44″N, 101°05′15.97″E	1263m	Mountainous area in a tropical rainforest region	Youle Jinuo; Mandarin Chinese	140	413	20	Male 11
								Female 9
Bapiao	22°02′49.53″N, 100°59′31.95″E	958m	Mountainous area in a tropical rainforest region	Youle Jinuo; Mandarin Chinese	143	408	22	Male 13
								Female 9
Luotelaozhai	22°03′31.35″N, 101°05′04.01″E	902m	Mountainous area in a tropical rainforest region	Youle Jinuo; Mandarin Chinese	37	150	10	Male 6
								Female 4
Puxilaozhai	22°01′00.60″N, 101°04′54.95″E	1056m	Mountainous area in a tropical rainforest region	Youle Jinuo; Mandarin Chinese	53	204	15	Male 8
								Female 7
Balai	21°55′36.77″N, 101°05′30.94″E	829m	Mountainous area in a tropical rainforest region	Youle Jinuo; Mandarin Chinese	95	374	20	Male 14
								Female 6
Zhaguo	22°07′01.95″N, 100°58′55.90″E	1031m	Mountainous area in a tropical rainforest region	Youle Jinuo; Mandarin Chinese	88	399	20	Male 13
								Female 7

All surveyed settlements were self-identified as Jinuo communities. No formal religion was declared by the informants

**Table 2:** Demographic features of informants

Demographic features		Number	Proportion (%)
Gender	Male	126	59.15
	Female	87	40.85
Age	18–30	15	7.04
	31–40	42	19.72
	41–50	70	32.86
	51–60	40	18.78
	61–70	32	15.02
	71 and above	14	6.57
Occupation	Farmers	150	70.42
	Civil servants	30	14.08
	Students	11	5.16
	Others	22	10.33

This study employed semi-structured interviews, participatory observation, key informant interviews, and focus group discussions. The main information collected during the interviews included the ethnobotanical names of medicinal plants, their habitats, the plant parts used, modes of preparation and administration, and their therapeutic functions. In addition, focus group discussions were conducted with local traditional Jinuo healers and villagers to examine the multiple use values of medicinal plants, their threatened status, conservation measures, and the transmission of traditional knowledge. To ensure the accuracy of plant identification and complete the list of medicinal plants, the recorded medicinal plant taxa were first identified according to the descriptions in Flora of China / iPlant and were then confirmed by Dr. Zhang Jiaqi. The nomenclature of all vascular plants was cross-checked against World Flora Online (https://www.worldfloraonline.org/) and standardized according to its currently accepted scientific names. The voucher specimen information was cited from the Herbarium (HITBC) of Xishuangbanna Tropical Botanical Garden, Chinese Academy of Sciences.

### Data analysis

Quantitative analysis of the survey data was conducted to evaluate the value of medicinal plants and the level of awareness of these plants within the local community, thereby helping to understand the potential of medicinal plants and associated traditional knowledge in the study area. In this study, the relative frequency of citation (RFC) was used to assess the commonly used or important medicinal plant species reported by informants for treating various diseases. The formula used is as follows:

$$RFC=FC\:/\:N $$

where FC represents the number of informants who mentioned the medicinal use of a given plant species, and N represents the total number of informants interviewed. RFC varies from 0 when nobody refers to the plant as useful to 1 when all informants mention the use of the species [34]. Generally, a higher RFC value indicates that the plant is more familiar to informants and is used more frequently, suggesting that the species has greater importance in the study area.

In addition, the Jaccard similarity index (JI) was used to compare the similarity of medicinal plant species composition between the present Jinuo study and previously published ethnobotanical studies. Species lists were standardized according to scientific names, and synonyms were unified before comparison. The JI was calculated as follows:

$$JI=C\,/\,\left({A+B-C}\right)\times100 $$

where A is the number of species recorded in the present study, B is the number of species recorded in the compared study, and C is the number of shared species [35]. Higher JI values indicate greater similarity in medicinal plant composition between the two studies.

## Results

### Diversity of medicinal plants within the study area

In the survey of Jinuo communities, a total of 226 medicinal plant species were recorded, belonging to 197 genera in 90 families (Table 3). Detailed information for each medicinal plant species, including its scientific name, Chinese name, Jinuo name, family, life form, habitat, parts used, modes of preparation and administration, therapeutic uses, and specimen number, is provided in Table 2. In terms of taxonomic composition, the dominant families were Fabaceae (16 species), Asteraceae (13), Lamiaceae (10), Euphorbiaceae (8), Zingiberaceae (7), Rubiaceae (7), and Solanaceae (7). In addition, 35 other families were represented by 2–6 species each, while 48 families were represented by only one species each (Table 4). In addition to widely distributed families across China, such as Fabaceae and Asteraceae, many species in dominant families such as Euphorbiaceae, Lamiaceae, Rubiaceae, and Zingiberaceae are mainly concentrated in tropical and subtropical regions, which to some extent reflects the distinct regional characteristics of medicinal plant use among the Jinuo people [36]. Xishuangbanna has a warm and humid climate with abundant rainfall and belongs to a typical tropical rainforest climatic zone. Therefore, the medicinal plants used locally are predominantly of tropical and subtropical distribution types. In addition, most traditional medicinal plants used by the Jinuo people are derived from wild resources. This pattern of using locally available materials is closely related to local medical conditions, the natural environment, and traditional ethnic lifestyles.

**Table 3:** Inventory of medicinal plants used by Jinuo people

Scientific name	Chinese name	Jinuo name (phonetic)	Family	Life form	Ha-bit	Parts used	Modes of preparation and administration	Therapeutic uses	RFC	Cited sources (HITBC)
*Abelmoschus moschatus* Medicus	黄葵	Bupaile	Malvaceae	Herb	Bot	R, L, Fr	Decoct in water and take orally; boil in water and wash externally	Dysentery, burns and scalds	0.17	HITBC0068528
*Abutilon indicum* (L.) Sweet	磨盘草	Shengsheng-xiele	Malvaceae	Herb	Bot	W	Decoct in water and take orally	Contraception, abortion, gonorrhoea	0.06	HITBC0030843
*Achyranthes aspera *L	土牛膝	Shengniang-pasa	Amaranthaceae	Herb	Wil	W	Decoct in water and take orally	Common cold, fever, tonsillitis, malaria	0.73	HITBC0026280
*Acmella paniculata* (Wall. ex DC.) R. K. Jansen	金纽扣	Zekezele	Asteraceae	Herb	Wil	W	Chew and hold against the affected area; decoct in water and take orally	Toothache, fracture	0.37	HITBC0060070
*Acorus calamus* L	菖蒲	Yichuo bubu	Acoraceae	Herb	Wil	W	Scrape into powder and take with boiling water; pound and apply as a poultice	Indigestion, abdominal pain, high fever, traumatic injuries, rheumatic bone pain	0.20	HITBC0023758
*Adenanthera microsperma* Teijsm. & Binn	海红豆	Tesheng	Fabaceae	Tree	Bot	Se	Roast the seeds over low heat until yellow, grind them, and take with boiling water	Dermatitis	0.21	HITBC0079382
*Ageratum conyzoides* L	藿香蓟	Miaonai	Asteraceae	Herb	Wil	W	Decoct in water and take orally	Common cold, fever, headache	0.34	HITBC0059497
*Agrimonia pilosa* Ledeb	龙牙草	Padeqieke	Rosaceae	Herb	Wil	W	Decoct in water and take orally	Irregular menstruation, hematuria	0.08	HITBC0029236
*Alangium kurzii* Craib	毛八角枫	Shede	Cornaceae	Tree	Wil	K	Decoct in water and take orally; grind dried leaves into powder and apply to the affected area	Pediatric bronchitis, cough, traumatic bleeding	0.02	HITBC0078123
*Alocasia odora* (Roxb.) K. Koch	海芋	Deyang	Araceae	Herb	Bot	Rh	Decoct in water and take orally	Skin itching, rheumatoid arthritis, snake and insect bites, hernia	0.59	HITBC0026773
*Alpinia blepharocalyx* K. Schum	云南草蔻	Miedu	Zingiberaceae	Herb	Bot	R	Decoct in water and take orally	Dyspepsia, stomachache	0.20	HITBC0069283
*Alpinia galanga* (L.) Willd	红豆蔻	Miepi	Zingiberaceae	Herb	Bot	R	Decoct in water and take orally	Dyspepsia	0.11	HITBC0026559
*Alstonia scholaris* (L.) R. Br	糖胶树	Jiejiao	Apocynaceae	Tree	Wil	B	Decoct in water and take orally	Resolving phlegm and relieving cough, chronic bronchitis, whooping cough	0.61	HITBC0078692
*Amomum maximum* Roxb	九翅豆蔻	Mieqie	Zingiberaceae	Herb	Bot	R	Decoct in water and take orally	Dyspepsia, stomach illness	0.13	HITBC0026749
*Amomum villosum* var. *xanthioides* (Wall. ex Baker) T. L. Wu & S. J. Chen	缩砂密	Miemi	Zingiberaceae	Herb	Bot	Fr,R	Decoct in water and take orally	Stomachache, diarrhea	0.60	HITBC0077176
*Amorphophallus konjac *K. Koch	魔芋	Wazaoawu	Araceae	Herb	Bot	Rh	Roast until warm and apply externally to the affected area; decoct in water and take orally	Traumatic injuries, boils, hernia, indigestion, unexplained swelling and sores	0.59	HITBC0079633
*Angiopteris caudatiformis* Hieron	披针观 音座莲	Demao	Marattiaceae	Herb	Wil	R,St	Decoct in water and take orally	Stomachache, enteritis, dysentery	0.03	HITBC0024421
*Anneslea fragrans* Wall	茶梨	Mazao-zhaimo	Pentaphylacaceae	Tree	Wil	B	Decoct in water and take orally; use corn liquor as a binder and apply as a poultice	Common cold, high fever, traumatic injury	0.12	HITBC0077586
*Anoectochilus roxburghii* (Wall.) Lindl	金线兰	Bieqiemiao	Orchidaceae	Herb	Wil	W	Decoct in water and take orally	Kidney stones, gallstones, pain from rheumatism	0.12	HITBC0056832
*Aralia decaisneana* Hance	台湾毛楤木	Taibo-paila	Araliaceae	Shrub	Wil	R	Decoct in water and take orally	Hepatitis, nephritis, rheumatism	0.38	HITBC030414
*Archidendron clypearia* (Jack) I. C. Nielsen	猴耳环	Yamiaobo	Fabaceae	Tree	Wil	L	Boil in water and wash externally; pound and apply as a poultice	Eye diseases, pinkeye	0.03	HITBC0030183
*Ardisia crenata* Sims	朱砂根	Jiangpujiang-yapami	Primulaceae	Shrub	Wil	R, L	Decoct in water and take orally; pound and apply externally	Cardiodynia, stomachache, traumatic injury, pain from rheumatism	0.17	HITBC0108641
*Ardisia solanacea* Roxb	酸薹菜	Jiangpujiang-yapamao	Primulaceae	Shrub	Wil	R, L	Pound and apply as a poultice	Osteoproliferation	0.09	HITBC0062685
*Areca catechu* L	槟榔	Maile	Arecaceae	Tree	Bot	Fr	Decoct in water and take orally	Pediatric bronchitis, dyspepsia, dysentery	0.32	HITBC0057782
*Arenga caudata* (Lour.) H. E. Moore	双籽棕	Miaola	Arecaceae	Shrub	Wil	L	Decoct in water and take orally	Used for abnormal uterine or vaginal bleeding, menorrhagia and pulmonary tuberculosis	0.04	HITBC0023843
*Argentina lineata* (Trevir.) Soják	西南蕨麻	Pawopu- lupami	Rosaceae	Herb	Wil	W	Decoct in water and take orally	Enteritis, dysentery	0.09	HITBC0027327
*Argyreia acuta* Lour	白鹤藤	Hemaozuolu	Convolvulaceae	Shrub	Wil	R,St	Decoct in water and take orally; boil in water and wash externally	Nephritis, pain from rheumatism and chronic bronchitis, eczema	0.08	HITBC0077807
*Artemisia argyi* H. Lév. & Vaniot	艾	Didi	Asteraceae	Herb	Wil	W	Boil in water and wash externally	Decoction used for external washing for eczema, skin itch and cough, with hemostatic effects	0.69	HITBC0070372
*Arundo donax* L	芦竹	Wulao	Poaceae	Herb	Wil	St	Decoct in water and take orally	Hepatitis	0.13	HITBC0024202
*Asparagus cochinchinensis* (Lour.) Merr	天门冬	Wutegasa	Asparagaceae	Herb	Wil	R	Decoct in water and take orally	Pulmonary tuberculosis, cough, bronchitis	0.24	HITBC0062729
*Baccaurea ramiflora* Lour	木奶果	Shengchi	Phyllanthaceae	Tree	Bot	R	Decoct in water and take orally	Dysentery	0.22	HITBC0058507
*Biancaea sappan* (L.) Tod	苏木	Laijie	Fabaceae	Tree	Bot	H	Decoct in water and take orally	Amenorrhea, postpartum abdominal pain due to blood stasis	0.16	HITBC0060797
*Bidens pilosa* L	鬼针草	Shengniang-baicuo	Asteraceae	Herb	Wil	W	Decoct in water and take orally; pound and apply externally	Upper respiratory tract infection, swollen sore throat, dyspepsia, traumatic injury	0.31	HITBC0058468
*Blumea* *balsamifera *(L.) DC	艾纳香	Busi	Asteraceae	Herb	Wil	R, L	Decoct in water and take orally	Toothache, hepatitis	0.44	HITBC0058734
*Blumea lanceolaria* (Roxb.) Druce	千头艾纳香	Lemao-shuomi	Asteraceae	Herb	Wil	W	Decoct in water and take orally; pound and apply externally	Contraception, traumatic injury, fracture	0.30	HITBC0061624
*Bombax ceiba* L	木棉	Liebie	Malvaceae	Tree	Bot	B, R	Pound and apply externally	Fracture, traumatic injury, furuncle	0.77	HITBC0032597
*Bryophyllum pinnatum* (L. f.) Oken	落地生根	Cegelabo- duo	Crassulaceae	Herb	Bot	W	Pound and apply externally	Mumps, mastitis, scalds, fracture	0.18	HITBC0030809
*Buddleja asiatica* Lour	白背枫	Hemi	Scrophulariaceae	Shrub	Bot	R	Decoct in water and take orally	Febrile convulsion	0.08	HITBC0059500
*Callicarpa bodinieri* H. Lév	紫珠	Qinuomiaoji	Lamiaceae	Shrub	Wil	R, L	Decoct in water and take orally	Abnormal uterine or vaginal bleeding, irregular menstruation, postpartum abdominal pain due to blood stasis	0.23	HITBC0058714
*Callicarpa macrophylla* Vahl	大叶紫珠	Luobubiao	Lamiaceae	Shrub	Wil	B, R	Decoct in water and take orally	Irregular menstruation, amenorrhea, dysmenorrhea	0.49	HITBC0071104
*Camphora glandulifera* (Wall.) Nees	云南樟	Guomao	Lauraceae	Tree	Bot	B	Decoct in water and take orally	Common cold, heatstroke, stomachache, diarrhea	0.81	HITBC0069157
*Canavalia gladiata* (Jacq.) DC	刀豆	Yaobiao	Fabaceae	Herb	Bot	Se, R	Decoct in water and take orally; pound and apply externally	Stomachache, traumatic injury	0.13	HITBC0011336
*Carex baccans* Nees	浆果薹草	Chenixiu	Cyperaceae	Herb	Bot	R	Decoct in water and take orally	Kidney stones, gallstones	0.11	HITBC0075931
*Caryota maxima* Blume ex Mart	鱼尾葵	Yaocai	Arecaceae	Tree	Wil	R, L	Decoct in water and take orally; pound and apply externally	Common cold, fever, cough, pulmonary tuberculosis, fracture, traumatic injury	0.40	XSBN001810
*Castanopsis argyrophylla* King ex Hook. f	银叶锥	Tupa	Fagaceae	Tree	Wil	R	Decoct in water and take orally	Enteritis, diarrhea	0.26	HITBC0027749
*Celosia cristata *L	鸡冠花	Yaoxieabo	Amaranthaceae	Herb	Bot	Fl	Decoct in water and take orally	Dysentery, uterine bleeding, excessive leukorrhea	0.39	HITBC0053443
*Centella asiatica* (L.) Urban	积雪草	Mijiupamao	Apiaceae	Herb	Wil	W	Decoct in water and take orally	Common cold, tonsillitis, hepatitis, enteritis, gallstone, snake bites, furuncle	0.77	HITBC0076069
*Chloranthus erectus* (Buch-Ham.) Verdc	鱼子兰	Mipacelu	Chloranthaceae	Shrub	Wil	W	Decoct in water and take orally; pound and apply externally	Toothache, fracture, traumatic injury, arthritis	0.18	HITBC0070188
*Chloranthus holostegius* (Hand.-Mazz.) S. J. Pei & Shan	全缘金粟兰	Abomire	Chloranthaceae	Herb	Wil	W	Decoct in water and take orally; pound and apply externally	Traumatic injury, joint pain, irregular menstruation	0.20	HITBC0030863
*Cibotium barometz* (L.) J. Sm	金毛狗	Debademao	Cibotiaceae	Herb	Bot	Rh	Decoct in water and take orally; pound and apply externally	Lower back and leg pain, rheumatic joint pain, bleeding from traumatic injuries	0.09	HITBC0068839
*Cinnamomum bejolghota* (Buch.-Ham.) Sweet	钝叶桂	Abopakei	Lauraceae	Tree	Wil	B	Decoct in water and take orally	Stomachache, rheumatic bone pain, fracture reduction	0.64	HITBC0028387
*Cinnamomum cassia* (L.) D. Don	肉桂	Pakei	Lauraceae	Tree	Cul	B, L, Fr	Decoct in water and take orally	Rheumatic joint pain, traumatic injury, fracture reduction, diarrhea	0.68	HITBC0069648
*Cissus repens* Lam	白粉藤	Nengyou-nengya	Vitaceae	Liana	Wil	St,L	Decoct in water and take orally; boil in water and wash externally	Rheumatic pain, eczema, venomous snake bite, sore	0.19	HITBC0062732
*Citrus maxima* (Burm.) Merr	柚	Majin	Rutaceae	Tree	Cul	L,Sk	Decoct in water and take orally	Stomachache, tonsillitis, pharyngitis	0.55	HITBC0061534
*Clausena excavata* N. L. Burman	假黄皮	Yawo-shannai	Rutaceae	Shrub	Bot	R, L	Decoct in water and take orally; boil in water and wash externally	Common cold, malaria, acute gastroenteritis, eczema	0.16	HITBC0058450
*Clerodendrum* *japonicum* (Thunb.) Sweet	赪桐	Bieboabo	Lamiaceae	Shrub	Bot	R, L	Decoct in water and take orally; pound and apply externally	Rheumatism, pulmonary tuberculosis, cough, traumatic injuries, abscesses, boils	0.21	HITBC0062619
*Codonopsis javanica* (Blume) Hook. f	金钱豹	Lemagete	Campanulaceae	Liana	Bot	R	Stew 100 g of the root with chicken or pork for consumption, or use in a formula	Diarrhea, neurasthenia, uterine prolapse	0.17	HITBC0062645
*Colocasia antiquorum* Schott	野芋	Lajiebie-enyang	Araceae	Herb	Bot	Rh	Pound and apply externally	Used for traumatic bleeding	0.50	HITBC075319
*Crateva unilocularis* Buch.-Ham	树头菜	Gemulai	Capparaceae	Tree	Bot	B, L	Decoct in water and take orally	Hepatitis, malaria	0.15	HITBC0057392
*Cratoxylum cochinchinense* (Lour.) Blume	黄牛木	Dechiguo	Hypericaceae	Tree	Wil	L	Pound and apply externally	Traumatic injury	0.25	HITBC0058674
*Crinum asiaticum* var. *sinicum* (Roxb.ex Herb.) Baker	文殊兰	Xiaoqiao-gumao	Amaryllidaceae	Herb	Bot	L,St	Pound with other ingredients in a formula and apply to the affected area	Pharyngitis, traumatic injury, fracture	0.57	HITBC0023800
*Crotalaria albida* B. Heyne ex Roth	响铃豆	Gege- shengmi	Fabaceae	Herb	Bot	W	Decoct in water and take orally; pound and apply externally	Malaria, hepatitis, cystitis, tracheitis, pneumonia, gastroenteritis, carbuncle, mastitis	0.73	HITBC0068427
*Crotalaria assamica* Benth	大猪屎豆	Gegesheng- mao	Fabaceae	Herb	Wil	R	Decoct in water and take orally	Rheumatism, nephritis, cystitis, urethritis, tonsillitis, dyspepsia	0.12	HITBC0068910
*Cryptolepis buchananii* Schult	古钩藤	Ziduoaneng	Apocynaceae	Liana	Wil	St,L	Pound and apply externally	Eczema, venomous snake bite	0.11	HITBC0062517
*Curculigo capitulata* (Lour.) Kuntze	大叶仙茅	Wushe-palai	Hypoxidaceae	Herb	Bot	R,St	Decoct in water and take orally; pound, mix with brown sugar, and apply to the affected area	Cough, sores, abscess	0.15	HITBC0078146
*Curculigo orchioides* Gaertn	仙茅	Duopo-wuli	Hypoxidaceae	Herb	Bot	R	Decoct in water and take orally	Erectile dysfunction, chronic nephritis	0.11	HITBC0079789
*Curcuma longa* L	姜黄	Nieshe	Zingiberaceae	Herb	Bot	Rh	Decoct in water and take orally	Icteric hepatitis, irregular menstruation, amenorrhea, traumatic injury	0.35	HITBC0023648
*Curcuma phaeocaulis* Valeton	莪术	Mieleng	Zingiberaceae	Herb	Bot	Rh	Pound and apply externally	Dyspepsia	0.33	HITBC0030628
*Cycas pectinata* Griff	篦齿苏铁	Dezhe	Cycadaceae	Shrub	Bot	L,St	Decoct in water and take orally	Gastritis, gastric ulcer, pulmonary tuberculosis	0.12	HITBC0021736
*Cymbidium sinense* (Jack. ex Andr.) Willd	墨兰	Midaka	Orchidaceae	Herb	Bot	R, Fl	Decoct in water and take orally	Kidney stones	0.09	HITBC050948
*Cymbopogon citratus* (DC.) Stapf	柠檬草	Saka	Poaceae	Herb	Bot	W	Decoct in water and take orally	Headache, stomachache, abdominal pain, rheumatism, diarrhea	0.70	XSBN002059
*Cynoglossum amabile* Stapf & Drummond	倒提壶	Hechasheng-niang	Boraginaceae	Herb	Wil	W	Pound and apply externally	With anti-inflammatory effects	0.07	HITBC0070947
*Davallia griffithiana* Hook	杯盖阴石蕨	Duozhuoxu	Davalliaceae	Herb	Bot	St	Decoct in water and take orally; pound and apply externally	Nephritis, traumatic injury	0.08	HITBC0059051
*Dendrobium densiflorum* Lindl	密花石斛	Dashe	Orchidaceae	Herb	Bot	St	Decoct in water and take orally	Icteric hepatitis	0.13	HITBC050881
*Dendrobium nobile* Lindl	石斛	Dacai	Orchidaceae	Herb	Bot	St	Decoct in water and take orally	Pulmonary tuberculosis	0.42	HITBC064552
*Dianella ensifolia* (L.) DC	山菅兰	Ertabubu	Asphodelaceae	Herb	Bot	W	Decoct in water and take orally; mix with vinegar and apply to the affected area	Hepatitis, scrofula, lymphadenitis	0.31	HITBC0077948
*Dichroa febrifuga* Lour	常山	Mipapa-mao	Hydrangeaceae	Shrub	Wil	L	Decoct in water and take orally	Malaria, with anti-inflammatory effects	0.46	HITBC0059140
*Dichrocephala chrysanthemifolia* (Blume) DC	菊叶鱼眼草	Luogao-cipamao	Asteraceae	Herb	Wil	W	Pound and apply externally; boil in water and wash externally	Snake bites, dermatitis, eczema	0.26	HITBC0068784
*Dicranopteris ampla* Ching & P. S. Chiu	大芒萁	Buluona	Gleicheniaceae	Herb	Bot	R,St,L	Boil in water and wash externally	Lacquer allergy	0.17	HITBC0096736
*Dicranopteris pedata* (Houtt.) Nakaike	芒萁	buluocha	Gleicheniaceae	Herb	Bot	R	Decoct in water and take orally	Urinary tract infection and scalds	0.17	HITBC0059895
*Dioscorea bulbifera* L	黄独	Liewulaga	Dioscoreaceae	Liana	Bot	T	Pound and apply externally	Endemic goiter, scrofula	0.16	HITBC0071164
*Dracaena cambodiana* Pierre ex Gagnep	柬埔寨 龙血树	Yebo-dengle	Asparagaceae	Tree	Wil	H	Grind the purple heartwood, mix with liquor, and take orally; grind into powder and apply to the wound	Anemia, stopping bleeding	0.17	HITBC0069468
*Drymaria cordata* (L.) Willdenow ex Schultes	荷莲豆草	Mikuobubu	Caryophyllaceae	Herb	Wil	W	Decoct in water and take orally	Icteric hepatitis, chronic nephritis	0.16	HITBC0029107
*Elaeagnus conferta* Roxb	密花胡颓子	Henu	Elaeagnaceae	Shrub	Bot	R,Fr	Decoct in water and take orally	Pediatric bronchitis, enteritis, dysentery, loss of appetite	0.20	HITBC0062204
*Elephantopus scaber* L	地胆草	Raoge-luoci	Asteraceae	Herb	Wil	W	Decoct in water and take orally	Common cold, acute tonsillitis, pharyngitis, pertussis	0.31	HITBC0059448
*Eleusine indica* (L.) Gaertn	牛筋草	Miupilie	Poaceae	Herb	Wil	W	Decoct in water and take orally	Dyspepsia, enteritis, dysentery	0.65	HITBC0061963
*Eleutherococcus trifoliatus* (Linnaeus) S. Y. Hu	白簕	Ajiao-jike	Araliaceae	Shrub	Wil	W	Decoct in water and take orally; soak in liquor and take orally; pound and apply as a poultice	Rheumatoid arthritis, traumatic injury, fracture	0.41	HITBC0059945
*Elsholtzia blanda* Benth	四方蒿	Jiwoluoge	Lamiaceae	Herb	Wil	W	Decoct in water and take orally	Abnormal uterine or vaginal bleeding, chronic pyelonephritis, dyspepsia	0.70	HITBC0068813
*Elsholtzia rugulosa* Hemsl	野拔子	Lanai	Lamiaceae	Herb	Wil	W	Decoct in water and take orally	Abdominal pain, common cold, headache, dyspepsia, acute gastroenteritis, dysentery	0.23	HITBC0068485
*Embelia ribes* Burm. F	白花酸藤果	Chetuo	Primulaceae	Liana	Wil	R, L, St	Decoct in water and take orally	Eye diseases, enteritis, dysentery, diarrhea	0.14	HITBC0059124
*Entada phaseoloides* (L.) Merr	榼藤	Poneng-pamao	Fabaceae	Liana	Wil	St	Decoct in water and take orally; soak in liquor and take orally	Diarrhea, traumatic injury, rheumatism	0.23	HITBC0059666
*Equisetum arvense* L	问荆	Nebaituo-jielaimi	Equisetaceae	Herb	Bot	St,L	Pound and apply externally	Burns and scalds	0.07	HITBC0024419
*Erigeron canadensis *L	小蓬草	Wazao-galai	Asteraceae	Herb	Wil	W	Decoct in water and take orally	Contraception	0.22	HITBC0078139
*Eryngium foetidum* L	刺芹	Paji	Apiaceae	Herb	Wil	W	Decoct in water and take orally	Dysentery, common cold, measles, tracheitis, enteritis, diarrhea	0.21	HITBC0078171
*Erythrina subumbrans* (Hassk.) Merr	翅果刺桐	Jileiduolei	Fabaceae	Tree	Wil	B	Decoct in water and take orally	Nephritis, edema, pulmonary tuberculosis, bronchitis	0.17	HITBC0030595
*Eupatorium fortunei* Turcz	佩兰	Quele-suomi	Asteraceae	Herb	Wil	W	Decoct in water and take orally; pound and apply externally	Traumatic injury	0.29	HITBC0019511
*Euphorbia neriifolia* L	金刚纂	Maxituoke	Euphorbiaceae	Tree	Bot	R, L	Decoct in water and take orally	Emphysema, bronchitis, carbuncle and furuncle	0.23	HITBC0021152
*Euphorbia hirta* L	飞扬草	Ziduomiao	Euphorbiaceae	Herb	Wil	W	Boil in water and wash externally	Eczema, dermatitis, dermatophytosis	0.08	HITBC0078818
*Fagopyrum dibotrys* (D. Don) Hara	金荞麦	Geye	Polygonaceae	Herb	Wil	Rh,L	Decoct in water and take orally; soak in liquor and take orally; pound and apply externally	Dysentery, rheumatoid arthritis, fracture, traumatic injury	0.80	HITBC0060785
*Ficus auriculata* Lour	大果榕	Shengpu	Moraceae	Tree	Wil	L	Decoct in water and take orally	Rectal prolapse	0.18	HITBC0060186
*Ficus pandurata* Hance	琴叶榕	Wamao-duosui	Moraceae	Shrub	Bot	R, L	Decoct in water and take orally	Pain from rheumatism, malaria, dysmenorrhea	0.14	HITBC0108676
*Ficus semicordata* Buch.-Ham. ex Sm	鸡嗉子榕	Shengjiu	Moraceae	Tree	Wil	Sk	Decoct in water and take orally	Rectal prolapse	0.22	HITBC0078070
*Fissistigma polyanthum* (Hook. f. & Thomson) Merr	黑风藤	Miusamami	Annonaceae	Shrub	Wil	R,St	Decoct in water and take orally	Rheumatoid arthritis, traumatic injury, fracture, common cold	0.38	HITBC0035925
*Glochidion eriocarpum* Champ. ex Benth	毛果算盘子	Jieshe	Phyllanthaceae	Shrub	Wil	W	Decoct in water and take orally; boil in water and wash externally	Enteritis, skin itch	0.24	HITBC0059907
*Glycosmis pentaphylla* (Retz.) Corrêa	山小橘	Mazhuopule	Rutaceae	Tree	Bot	St,R,L	Decoct in water and take orally; pound and apply externally	Tracheitis, venomous snake bites, traumatic injury	0.62	HITBC0109852
*Gmelina arborea* Roxb	云南石梓	Lemie	Lamiaceae	Tree	Bot	B, L	Decoct in water and take orally; pound and apply externally	Rectal prolapse, fracture	0.65	HITBC0061098
*Gnetum montanum* Markgr	买麻藤	Lebai-shengmi	Gnetaceae	Liana	Bot	St	Decoct in water and take orally	Rheumatoid arthritis	0.07	HITBC0059115
*Guilandina minax* (Hance) G. P. Lewis	喙荚鹰叶刺	Yaji	Fabaceae	Liana	Bot	R, L, Se	Decoct in water and take orally; pound and apply externally	Common cold, rheumatoid arthritis, traumatic injury, fracture	0.06	HITBC0069628
*Gymnosporia acuminata* Hook. f	美登木	Ajiaolabo	Celastraceae	Shrub	Wil	L	Decoct in water and take orally	Loss of appetite	0.54	HITBC0069721
*Gynostemma pentaphyllum* (Thunb.) Makino	绞股蓝	Anenggeichi	Cucurbitaceae	Liana	Bot	W	Decoct in water and take orally; pound and apply as a poultice	Heat-clearing and detoxifying effects, traumatic injuries, resolving phlegm and relieving cough	0.85	HITBC0080032
*Hedyotis capitellata* Wall. ex G. Don	头状花耳草	Xianna	Rubiaceae	Herb	Wil	W	Decoct in water and take orally; pound and apply externally	Icteric hepatitis, fracture reduction	0.08	HITBC0029453
*Hedyotis hedyotidea* (DC.) Merr	牛白藤	Xiansu	Rubiaceae	Shrub	Wil	W	Pound and apply externally	Traumatic injury, fracture	0.08	HITBC0058392
*Hedyotis tenelliflora* Blume	纤花耳草	Paqi	Rubiaceae	Herb	Wil	W	Decoct in water and take orally	Traumatic injury, hernia, toothache	0.12	HITBC0034266
*Hellenia speciosa* (J. Koenig) S. R. Dutta	闭鞘姜	Baiqiekepo	Costaceae	Herb	Bot	W	Decoct in water and take orally; pound and apply externally	Urinary tract infection, edema, heart disease, nephritis, urticaria	0.14	HITBC0080057
*Heynea trijuga* Roxb	鹧鸪花	Zuoduobolie	Meliaceae	Tree	Wil	R	Decoct in water and take orally	Rheumatoid arthritis, tonsillitis	0.08	HITBC0058744
*Homalomena* *occulta *(Lour.) Schott	千年健	Lebo-yaotai	Araceae	Herb	Wil	Rh	Decoct in water and take orally; pound and apply externally	Rheumatic low back and leg pain, stomachache, gastroenteritis, traumatic injury and fracture	0.82	HITBC0023786
*Homonoia riparia* Lour	水柳	Miebang	Euphorbiaceae	Shrub	Wil	W	Decoct in water and take orally; pound and apply externally	Hemorrhoids, cystitis, cholecystitis, traumatic injury	0.14	HITBC0021187
*Houttuynia cordata* Thunb	蕺菜	Sangbie-daci	Saururaceae	Herb	Bot	W	Decoct in water and take orally	Tonsillitis, pneumonia, tracheitis, dysentery, mastitis	0.78	HITBC0109911
*Hypericum japonicum* Thunb. ex Murray	地耳草	Piaobeici	Hypericaceae	Herb	Wil	W	Decoct in water and take orally; pound and apply externally	Malaria, hepatitis, appendicitis, scabies	0.69	HITBC0060833
*Imperata cylindrica* (L.) P. Beauv	白茅	Yiqie	Poaceae	Herb	Wil	W	Decoct in water and take orally	Nephritis, edema, hypertension	0.44	HITBC0077401
*Iris tectorum* Maxim	鸢尾	Misai	Iridaceae	Herb	Bot	Rh	Pound and apply externally	Arthritis, traumatic injury, hepatitis	0.06	HITBC0023809
*Jatropha curcas* L	麻风树	Quanmilao	Euphorbiaceae	Tree	Bot	L, B	Pound and apply externally; boil in water and wash externally	Traumatic injury, fracture, skin itch, eczema	0.03	HITBC0071172
*Juncus effusus *L	灯芯草	Shetunadu	Juncaceae	Herb	Bot	W	Decoct in water and take orally	Malaria, urinary tract infection	0.75	HITBC0066146
*Kadsura heteroclita* (Roxb.) Craib	异形南 五味子	Abochetuo	Schisandraceae	Liana	Wil	R,St	Decoct in water and take orally; soak in liquor and take orally	Rheumatoid arthritis, common cold, bronchitis	0.08	HITBC0079621
*Kyllinga brevifolia* Rottb	短叶水蜈蚣	Shetududu-anv	Cyperaceae	Herb	Wil	W	Boil in water and wash externally, decoct in water and take orally	Allergy, rheumatic joint pain, dysentery	0.06	HITBC0061093
*Lablab purpureus *(L.) Sweet	扁豆	Biaonai	Fabaceae	Liana	Cul	L	Decoct in water and take orally	Used for upset stomach and emesis	0.19	HITBC0075964
*Laggera crispata* (Vahl) Hepper & J. R. I. Wood	翼齿六棱菊	Yaokuo-yaoniang	Asteraceae	Herb	Wil	W	Decoct in water and take orally	Common cold, heatstroke, stomatitis, malaria, fracture reduction	0.23	HITBC0070801
*Leucas ciliata* Hochst. ex Benth	绣球防风	Guoque-yaba	Lamiaceae	Herb	Wil	W	Decoct in water and take orally	Common cold, mumps, malaria	0.06	HITBC0078483
*Ligustrum lucidum* W. T. Aiton	女贞	Yasheng	Oleaceae	Tree	Wil	L	Chew fresh leaves and swallow	Pharyngitis, stomatitis	0.09	HITBC0058374
*Litsea pungens* Hemsl	木姜子	Shengbi	Lauraceae	Tree	Bot	R	Decoct in water and take orally	Irregular menstruation	0.10	HITBC0060543
*Livistona saribus* (Lour.) Merr. ex A. Chev	大叶蒲葵	Chupu	Arecaceae	Tree	Wil	L	Decoct in water and take orally	Nephritis, kidney stones, cystitis	0.24	XSBN002989
*Lobelia nummularia* Lam	铜锤玉带草	Mijiupasu	Campanulaceae	Herb	Wil	W	Soak in liquor and take orally	Traumatic injury, pain from rheumatism	0.10	HITBC0062056
*Lobelia seguinii* H. Lév. & Vaniot	西南山梗菜	Shengmao-kehai	Campanulaceae	Herb	Wil	W	Decoct in water and take orally	Rheumatoid arthritis, traumatic injury	0.07	HITBC0029541
*Lycopodium japonicum* Thunb	石松	Michimiu	Lycopodiaceae	Herb	Wil	W	Decoct in water and take orally	Pain from rheumatism, traumatic injury	0.02	HITBC0069048
*Lygodium flexuosum* (L.) Sw	曲轴海金沙	Dewuxing	Lygodiaceae	Liana	Wil	W	Decoct in water and take orally	Hepatitis, dysentery, chronic nephritis	0.14	HITBC0058467
*Lygodium japonicum* (Thunb.) Sw	海金沙	Dewuli	Lygodiaceae	Herb	Wil	W	Decoct in water and take orally	Fever, dysentery, enteritis, urinary tract infection, urinary stones	0.22	HITBC0061168
*Lysimachia capillipes* Hemsl	细梗香草	Rimi	Primulaceae	Herb	Wil	W	Decoct in water and take orally; pound and apply externally	Rheumatic joint pain	0.08	HITBC0068041
*Lysimachia foenum-graecum* Hance	灵香草	Remi	Primulaceae	Herb	Bot	W	Decoct in water and take orally	Toothache, rheumatic bone pain, tracheitis, cough, common cold, irregular menstruation	0.10	HITBC0027881
*Maesa indica* (Roxb.) A. DC	包疮叶	Abolekei	Primulaceae	Shrub	Wil	St,L	Decoct in water and take orally; pound and apply externally	Icteric hepatitis, furuncle	0.23	HITBC0059265
*Malaxis monophyllos* (L.) Sw	原沼兰	MaiFang	Orchidaceae	Herb	Wil	W	Decoct in water and take orally	Hepatitis, gastric ulcer	0.08	HITBC0035942
*Mallotus barbatus* (Wall. ex Baill.) Müll. Arg	毛桐	Naishengchi	Euphorbiaceae	Tree	Wil	R,St,L	Decoct in water and take orally	Rheumatoid arthritis, urethritis	0.06	HITBC0058495
*Mallotus philippensis* (Lam.) Muell. Arg	粗糠柴	Cheshnegta	Euphorbiaceae	Tree	Wil	R,St,L,B	Decoct in water and take orally; pound and apply externally	Headache, diarrhea, traumatic injury	0.03	HITBC0057956
*Maoutia puya* (Hook.) Wedd	水丝麻	Zike	Urticaceae	Shrub	Wil	W	Decoct in water and take orally	Common cold with fever, hypermenorrhea, pain from rheumatism	0.03	HITBC0062677
*Markhamia stipulata* var. *kerrii* Sprague	毛叶猫尾木	Hemaoduo-miabiao	Bignoniaceae	Tree	Wil	L	Decoct in water and take orally	Common cold, fever	0.24	HITBC0026524
*Melastoma malabathricum* L	印度野牡丹	Baibai-laoniang	Melastomataceae	Shrub	Bot	W	Decoct in water and take orally	Enteritis, diarrhea, dysentery	0.18	HITBC0058000
*Melicope pteleifolia* (Champ. ex Benth.) Hartley	三桠苦	Pakepami	Rutaceae	Tree	Wil	W	Decoct in water and take orally	Rheumatoid arthritis, sciatica, influenza	0.63	HITBC0058365
*Melodinus cochinchinensis* (Loureiro) Merrill	思茅山橙	Qiene	Apocynaceae	Liana	Wil	R,Fr	Decoct in water and take orally	Gastric ulcer, infantile meningitis, fracture	0.28	HITBC0077478
*Michelia gioii* (A. Chev.) Sima & H. Yu	香子含笑	Shaomu	Magnoliaceae	Tree	Wil	Se	Decoct in water and take orally	Dyspepsia	0.04	HITBC0035747
*Mirabilis jalapa* L	紫茉莉	Weiweiabo	Nyctaginaceae	Herb	Bot	R, L	Pound and apply externally	Edema, mumps, mastitis, furuncle	0.33	HITBC0070744
*Momordica charantia* L	苦瓜	Anenggekao	Cucurbitaceae	Herb	Bot	Fr	Decoct in water and take orally; pound and apply externally	Hepatitis, dysentery, with heat-clearing and detoxifying effects, snake and insect bites	0.72	HITBC0071189
*Munronia pinnata* (Wall.) W. Theob	羽状地黄连	Xipacui	Meliaceae	Shrub	Wil	R	Soak in liquor and take orally; rub externally	Malaria, traumatic injury	0.08	HITBC0031194
*Musa acuminata* Colla	小果野蕉	Lanai	Musaceae	Herb	Wil	Fr,Fl	Pound and apply externally	Heart disease, fracture, traumatic injury	0.20	HITBC107266
*Mussaenda pubescens* W. T. Aiton	玉叶金花	Anengai-bulao	Rubiaceae	Shrub	Wil	R,St	Decoct in water and take orally	Pertussis, pulmonary disease	0.77	HITBC0059106
*Nicotiana tabacum* L	烟草	Yaokuo	Solanaceae	Herb	Cul	W	Pound and apply externally	Eye diseases, insect bites	0.07	HITBC0078909
*Oenanthe javanica *(Bl.) DC	水芹	Padepamao	Apiaceae	Herb	Bot	W	Decoct in water and take orally; eat raw	Used for hypertension, urinary tract infection, abdominal pain	0.57	HITBC0062717
*Oroxylum indicum* (L.) Kurz	木蝴蝶	Bonashuoluo	Bignoniaceae	Tree	Wil	Se,B	Infuse in water and take orally	Hepatitis, bronchitis, gastric ulcer	0.73	HITBC0078040
*Papaver somniferum *L	罂粟	Yangyan	Papaveraceae	Herb	Wil	L	Decoct in water and take orally	Malaria, high fever	0.49	HITBC0047479
*Paris polyphylla* Sm	七叶一枝花	Akeilici	Melanthiaceae	Herb	Bot	R	Decoct in water and take orally; mix with vinegar and apply to the affected area	Pneumonia, tonsillitis, nephritis, gastritis, furuncle, arthritis, venomous snake bites	0.72	HITBC0028445
*Pedilanthus tithymaloides* (L.) Poit	红雀珊瑚	Maxituoluo	Euphorbiaceae	Shrub	Cul	St	Pound and apply externally	Traumatic injury	0.02	HITBC0032801
*Persicaria chinensis* (L.) H. Gross	火炭母	Gelaiche	Polygonaceae	Herb	Wil	St,L	Decoct in water and take orally	Dysentery, enteritis, dyspepsia, abdominal pain, emesis	0.43	HITBC0059911
*Phlogacanthus curviflorus* (Wall.) Nees	火焰花	Yuexie-pamao	Acanthaceae	Shrub	Wil	R	Decoct in water and take orally	Malaria, heat-clearing and detoxifying effects	0.47	HITBC0059356
*Pholidota chinensis* Lindl	石仙桃	Yaqieyene	Orchidaceae	Herb	Bot	W	Pound and apply externally	Tracheitis, pulmonary heart disease, chronic osteomyelitis	0.07	HITBC0067860
*Phrynium rheedei* Suresh & Nicolson	柊叶	Paipajiene	Marantaceae	Herb	Wil	R,St	Decoct in water and take orally	Nephritis, cystitis, diuresis, dysentery	0.67	HITBC0077535
*Phyllanthus emblica* L	余甘子	Shengchao	Phyllanthaceae	Tree	Bot	R, L, B	Decoct in water and take orally; boil in water and wash externally	Common cold, headache, enteritis, diarrhea, eczema	0.82	HITBC0061459
*Phyllanthus urinaria* L	叶下珠	Yuchuo-shengchuan	Phyllanthaceae	Herb	Wil	W	Decoct in water and take orally; pound and apply externally	Fever, hepatitis and dysentery; applied externally for snake bites, with detoxifying effects	0.84	HITBC0062740
*Pinus yunnanensis* Franch	云南松	Tesu	Pinaceae	Tree	Wil	B	Soak in liquor and take orally; decoct in water and take orally	Pain from rheumatism, cough	0.18	HITBC0061060
*Piper boehmeriifolium* (Miq.) Wall. ex C. DC	苎叶蒟	Peigecelu	Piperaceae	Shrub	Wil	R, L	Decoct in water and take orally	Rheumatoid arthritis, pain from rheumatism, common cold	0.15	HITBC0077206
*Plantago asiatica* L	车前	Ahuoyukui	Plantaginaceae	Herb	Wil	W	Decoct in water and take orally	Fever, urinary obstruction, urinary stones	0.87	HITBC0058387
*Plumbago zeylanica* L	白花丹	Budeleci	Plumbaginaceae	Shrub	Wil	R, L	Decoct in water and take orally; pound and apply externally	Rheumatism, rheumatoid arthritis, traumatic injury	0.69	HITBC0076086
*Pogonatherum crinitum* (Thunb.) Kunth	金丝草	Yaobuyao	Poaceae	Herb	Wil	W	Infuse in water and drip into the ear; pound and apply externally; decoct in water and take orally	Otitis media, traumatic bleeding, diabetes	0.37	HITBC0058438
*Pogostemon glaber* Benth	刺蕊草	Buheboci	Lamiaceae	Herb	Wil	W	Decoct in water and take orally	Allergy, measles	0.06	HITBC0068293
*Polygonatum cirrhifolium* (Wall.) Royle	卷叶黄精	Hepiduo-gele	Asparagaceae	Herb	Bot	R,St	Decoct in water and take orally	Used for swollen sore throat, cough and postpartum deficiency	0.38	HITBC0060660
*Premna szemaoensis* C. Pei	思茅豆腐柴	Mamopane	Lamiaceae	Tree	Wil	B	Pound and apply as a poultice	Fracture and traumatic injury	0.69	HITBC0028716
*Prunus persica* (L.) Batsch	桃	Shengyan	Rosaceae	Tree	Bot	B, L	Boil the bark in water and wash externally; roast the leaves until soft, pound, and apply to the affected area	Skin itch	0.07	HITBC0065575
*Psidium guajava* L	番石榴	Magui	Myrtaceae	Tree	Bot	L,Fr	Decoct in water and take orally; chew the fresh material and swallow; pound and apply externally	Diarrhea, migraine	0.83	HITBC0070661
*Psophocarpus tetragonolobus* (L.) DC	四棱豆	Heqiule-ciabiao	Fabaceae	Herb	Cul	R	Decoct in water and take orally	Rheumatoid arthritis	0.13	HITBC0013986
*Pteridium revolutum* (Blume) Nakai	毛轴蕨	Dechi	Dennstaedtiaceae	Herb	Wil	R	Decoct in water and take orally	Impotence	0.13	HITBC0029858
*Pueraria montana* var. *lobata*	葛	Qieneng	Fabaceae	Liana	Bot	R	Decoct in water and take orally	Abdominal pain	0.76	HITBC0021713
*Punica granatum* L	石榴	Ajiumie-sheng	Lythraceae	Tree	Bot	L	Decoct in water and take orally; chew the fresh material and swallow	Enteritis, dysentery	0.14	HITBC0060576
*Pyrrosia piloselloides* (L.) M. G. Price	抱树莲	Dabulu	Polypodiaceae	Herb	Wil	W	Decoct in water and take orally	Cough, lymph node tuberculosis	0.18	XSBN000067
*Rauvolfia verticillata* (Lour.) Baill	萝芙木	Niangda	Apocynaceae	Shrub	Wil	R,St,L	Decoct in water and take orally; pound and apply as a poultice	Hypertension, abdominal pain, traumatic injury	0.08	HITBC0058249
*Remusatia* *vivipara *(Lodd.) Schott	岩芋	Jichui-xuechui	Araceae	Herb	Wil	R,St	Pound with vinegar and apply externally	Mastitis, traumatic injury, carbuncle and furuncle, skin and soft tissue infections, rheumatoid joint pain	0.06	HITBC0056573
*Rhaphidophora decursiva* (Roxb.) Schott	爬树龙	Hegaolao-paqie	Araceae	Liana	Wil	St	Pound with other ingredients in a formula and apply to the affected area	Heat-clearing and detoxifying effects, traumatic injuries, resolving phlegm and relieving cough	0.08	HITBC0026053
*Rhus chinensis *Mill	盐麸木	Shengmao	Anacardiaceae	Tree	Wil	R, B, L	Decoct in water and take orally; pound and apply externally	Dysentery, fracture, traumatic injury	0.23	HITBC0059423
*Ricinus communis* L	蓖麻	Nekeilaokei	Euphorbiaceae	Herb	Bot	Se,L	Decoct in water and take orally; pound and apply externally	Traumatic injury, headache	0.71	HITBC0071163
*Rorippa indica* (L.) Hiern	蔊菜	Gemaoletuo	Brassicaceae	Herb	Wil	W	Decoct in water and take orally	Epilepsy, icterohepatitis	0.19	HITBC0079535
*Rotheca* *serrata *(L.) Steane & Mabb	三对节	Yashangnai	Lamiaceae	Shrub	Bot	R,L,B	Decoct in water and take orally; pound and apply warm to the affected area	Tonsillitis, pharyngitis, hepatitis, malaria, pain from rheumatism, fracture, traumatic injury	0.08	HITBC0034671
*Rubus ellipticus* var. *obcordatus* (Franch.) Focke	栽秧藨	Shenglao-laone	Rosaceae	Shrub	Wil	R	Decoct in water and take orally	Dysentery, diarrhea, toothache, swollen sore throat	0.21	HITBC0057916
*Rumex nepalensis* Spreng	尼泊尔酸模	Luoche	Polygonaceae	Herb	Wil	R, L	Decoct in water and take orally; pound and apply externally	Scabies, traumatic injury, diuretic effects	0.47	HITBC0035485
*Sambucus adnata* Wall. ex DC	血满草	Keci	Viburnaceae	Herb	Wil	R, L	Decoct in water and take orally; pound and apply as a poultice	Hepatitis, fracture	0.83	HITBC0062395
*Schima wallichii* (DC.) Korth	西南木荷	Shengsa	Theaceae	Tree	Bot	L	Decoct in water and take orally	Dysentery, epistaxis	0.02	HITBC0026277
*Schisandra neglecta* A. C. Sm	滇藏五味子	Quexiu-naishao	Schisandraceae	Liana	Wil	R	Decoct in water and take orally; soak in liquor and take orally	Common cold, headache, fever, rheumatoid arthritis	0.07	HITBC0039970
*Schizomussaenda henryi* (Hutch.) X. F. Deng & D. X. Zhang	裂果金花	Ebuluo	Rubiaceae	Shrub	Wil	L, R	Decoct in water and take orally	Tracheitis, cough, respiratory tract infection	0.23	HITBC0075663
*Scleromitrion diffusum* (Willd.) R. J. Wang	白花蛇舌草	Luoche	Rubiaceae	Herb	Wil	W	Decoct in water and take orally	Tumors, pharyngitis, traumatic injury	0.19	HITBC0079994
*Scoparia dulcis *L	野甘草	Pamiqiechi	Plantaginaceae	Herb	Wil	W	Decoct in water and take orally	Emphysema, cough, icteric hepatitis	0.36	HITBC0058666
*Selaginella uncinata* (Desv.) Spring	翠云草	Mimen	Selaginellaceae	Herb	Wil	W	Pound and apply externally	Rheumatism, traumatic injury	0.10	HITBC0060960
*Senna tora* (L.) Roxb	决明	Qibienajiao	Fabaceae	Herb	Bot	W	Decoct in water and take orally	Acute conjunctivitis, hepatitis	0.18	HITBC0075954
*Sida szechuensis* Matsuda	拔毒散	Beikou-lashao	Malvaceae	Shrub	Wil	W	Decoct in water and take orally; pound and apply externally	Amenorrhea, furuncle	0.06	HITBC0029210
*Sigesbeckia orientalis* L	豨莶	Zaikezaile	Asteraceae	Herb	Wil	W	Decoct in water and take orally	Malaria, acute hepatitis, traumatic bleeding	0.39	HITBC0078135
*Smilax ocreata* A. DC	抱茎菝葜	Qiemao	Smilacaceae	Shrub	Wil	R,St	Decoct in water and take orally	Skin allergy, neurasthenia, stomachache	0.05	HITBC0079354
*Solanum erianthum* D. Don	假烟叶树	Depai	Solanaceae	Tree	Wil	R	Decoct in water and take orally	Stomachache, abdominal pain	0.04	HITBC0058494
*Solanum melongena* L	茄	Makei	Solanaceae	Herb	Cul	Fr	Decoct in water and take orally	Diuresis, beriberi, toothache	0.10	HITBC0060371
*Solanum nigrum* L	龙葵	Geli	Solanaceae	Herb	Bot	W	Decoct in water and take orally; pound and apply externally	Common cold with fever, bronchitis, dysentery, venomous snake bites, furuncle	0.72	HITBC0061962
*Solanum spirale* Roxb	旋花茄	Zuojiao-shengkao	Solanaceae	Shrub	Bot	R	Decoct in water and take orally	Abdominal pain, malaria, common cold, fever, swollen sore throat	0.68	HITBC0109899
*Solanum torvum* Sw	水茄	Mujie-shengkao	Solanaceae	Shrub	Bot	Fr	Decoct in water and take orally; pound and apply externally	Traumatic injury	0.15	HITBC0059084
*Solanum virginianum* L	黄果茄	Makeiayao	Solanaceae	Herb	Wil	R	Decoct in water and take orally; stew with chicken for consumption	Common cold, fever, headache	0.77	HITBC107268
*Spatholobus suberectus* Dunn	密花豆	Nengnuo	Fabaceae	Liana	Wil	R	Decoct in water and take orally	Diarrhea, anemia, irregular menstruation, amenorrhea	0.25	HITBC0061107
*Spondias* *pinnata *(L. F.) Kurz	槟榔青	Kezeng	Anacardiaceae	Tree	Bot	B,Fr	Decoct in water and take orally	Common cold, dyspepsia	0.80	HITBC0062662
*Stephania delavayi* Diels	一文钱	Yikei-maojie	Menispermaceae	Liana	Wil	W	Decoct in water and take orally	Stomachache, enteritis, common cold, stomatitis, asthma	0.76	HITBC0067087
*Stephania epigaea* H. S. Lo	地不容	Wutuocaci	Menispermaceae	Liana	Bot	R	Decoct in water and take orally; pound and apply externally	Abnormal uterine or vaginal bleeding, stomachache, neurasthenia, traumatic injury, fracture	0.86	HITBC0026316
*Stereospermum colais* (Buch.-Ham. ex Dillwyn) Mabb	羽叶楸	Xuexie	Bignoniaceae	Tree	Wil	L	Pound and apply externally	Nerve headache	0.11	HITBC0079769
*Strobilanthes cusia* (Nees) Kuntze	板蓝	Mipapami	Acanthaceae	Herb	Bot	R, L	Pound and apply externally; decoct in water and take orally	Osteoproliferation, tonsillitis, common cold, stomatitis, mumps	0.77	HITBC0077607
*Tacca chantrieri* André	箭根薯	Shengke-palai	Dioscoreaceae	Herb	Wil	Rh,L	Decoct in water and take orally; pound with other ingredients in a formula and apply to the affected area	Pharyngitis, tonsillitis, pneumonia, malaria, stomach illness, traumatic injury	0.85	HITBC0079893
*Tadehagi triquetrum* (L.) Ohashi	葫芦茶	Yidepacai	Fabaceae	Herb	Bot	W	Decoct in water and take orally	Hepatitis, enteritis, dysentery, dyspepsia	0.68	HITBC0078035
*Tamarindus indica* L	酸豆	Qiubiao-azeng	Fabaceae	Tree	Bot	B	Decoct in water and take orally	Dysentery, diarrhea	0.24	HITBC0077178
*Tetrastigma planicaule* (Hook.) Gagnep	扁担藤	Nengtai	Vitaceae	Liana	Wil	St	Decoct in water and take orally	Pediatric bronchitis, neurasthenia, rheumatoid arthritis	0.19	HITBC0059133
*Tinospora crispa* (L.) Hook. f. & Thomson	波叶青牛胆	Maixinaineng	Menispermaceae	Liana	Bot	R,St	Decoct in water and take orally; pound and apply externally	Urethritis, rheumatism, snake and insect bites, traumatic injury, fracture	0.19	HITBC0079297
*Toddalia asiatica* (L.) Lam	飞龙掌血	Maiqiu-maile	Rutaceae	Liana	Wil	W	Decoct in water and take orally; soak in liquor and take orally	Traumatic injury, fracture	0.12	HITBC0059154
*Trevesia palmata* (Roxb.) Vis	刺通草	Taiyi	Araliaceae	Tree	Wil	R, L	Pound and apply as a poultice	Traumatic injury, fracture	0.35	HITBC0077956
*Uncaria scandens* (Sm.) Hutch	攀茎钩藤	Laqiwuyou	Rubiaceae	Liana	Wil	R,St,L	Decoct in water and take orally; boil in water and wash externally; pound and apply externally	Tracheitis, common cold, traumatic injury	0.18	HITBC0068873
*Verbena officinalis* L	马鞭草	Anaiduo	Verbenaceae	Herb	Wil	W	Decoct in water and take orally; boil in water and wash externally	Abdominal pain, abnormal uterine or vaginal bleeding, malaria	0.79	HITBC0062712
*Vernonia parishii* Hook. f	滇缅斑鸠菊	Busi-kaopao	Asteraceae	Tree	Wil	W	Decoct in water and take orally	Common cold, fever, postpartum deficiency, rheumatic bone pain, hepatitis	0.16	HITBC0058430
*Viburnum cylindricum* Buch.-Ham. ex D. Don	水红木	Latupazha	Viburnaceae	Tree	Wil	R, L	Decoct in water and take orally; pound and apply as a poultice	Hepatitis, cough, bronchitis and applied externally for scalds	0.74	HITBC0079759
*Zanthoxylum armatum* DC	竹叶花椒	Zebi	Rutaceae	Tree	Bot	R, L	Decoct in water and take orally	Irregular menstruation, common cold, headache, rheumatic low back and leg pain	0.51	HITBC0058082
*Zehneria japonica* (Thunb.) H. Y. Liu	马瓟儿	Bajuebayao	Cucurbitaceae	Herb	Wil	R, L	Decoct in water and take orally; pound and apply externally	Cystitis, traumatic injury	0.15	HITBC0071190
*Zingiber officinale* Roscoe	姜	Chaoke	Zingiberaceae	Herb	Cul	Rh	Decoct in water and take orally	Common cold, stomach cold, emesis	0.60	HITBC048852

Bot: both wild and cultivated, Cul: cultivated, Wil: wild; B: bark, Fl: flower, Fr: fruit, H: heartwood, K: kernel, L: leaf, R: root, Rh: rhizome, Se: seed, Sk: skin of fruit, St: stem, T: tuber, and W: whole plant

**Table 4:** Distribution of medicinal plants of the Jinuo people in each family

Category (number of medicinal plants)	Family
Greater than or equal to 10	Fabaceae (16), Asteraceae (13), Lamiaceae (10)
5 to 9	Euphorbiaceae (8), Zingiberaceae (7), Rubiaceae (7), Solanaceae (7), Primulaceae (6), Orchidaceae (6), Araceae (6), Rutaceae (6), Poaceae (5)
2 to 4	Apocynaceae (4), Malvaceae (4), Rosaceae (4), Phyllanthaceae (4), Lauraceae (4), Arecaceae (4), Menispermaceae (3), Cucurbitaceae (3), Campanulaceae (3), Polygonaceae (3), Apiaceae (3), Moraceae (3), Asparagaceae (3), Araliaceae (3), Bignoniaceae (3), Plantaginaceae (2), Lygodiaceae (2), Hypericaceae (2), Chloranthaceae (2), Acanthaceae (2), Gleicheniaceae (2), Meliaceae (2),Vitaceae (2), Anacardiaceae (2), Cyperaceae (2), Dioscoreaceae (2), Viburnaceae (2), Schisandraceae (2), Hypoxidaceae (2), Amaranthaceae (2)
1	Asphodelaceae, Musaceae, Smilacaceae, Plumbaginaceae, Costaceae,Acoraceae, Juncaceae, Annonaceae, Davalliaceae, Marattiaceae, Piperaceae, Elaeagnaceae, Cibotiaceae, Crassulaceae, Selaginellaceae, Fagaceae, Melanthiaceae, Verbenaceae, Gnetaceae, Magnoliaceae, Oleaceae, Equisetaceae, Lythraceae, Saururaceae, Theaceae, Capparaceae, Cornaceae, Brassicaceae, Lycopodiaceae, Amaryllidaceae, Caryophyllaceae, Polypodiaceae, Pinaceae, Cycadaceae, Myrtaceae, Dennstaedtiaceae, Celastraceae, Pentaphylacaceae, Hydrangeaceae, Scrophulariaceae, Convolvulaceae, Urticaceae, Melastomataceae, Papaveraceae, Iridaceae, Marantaceae, Boraginaceae, Nyctaginaceae

**Fig. 4:**
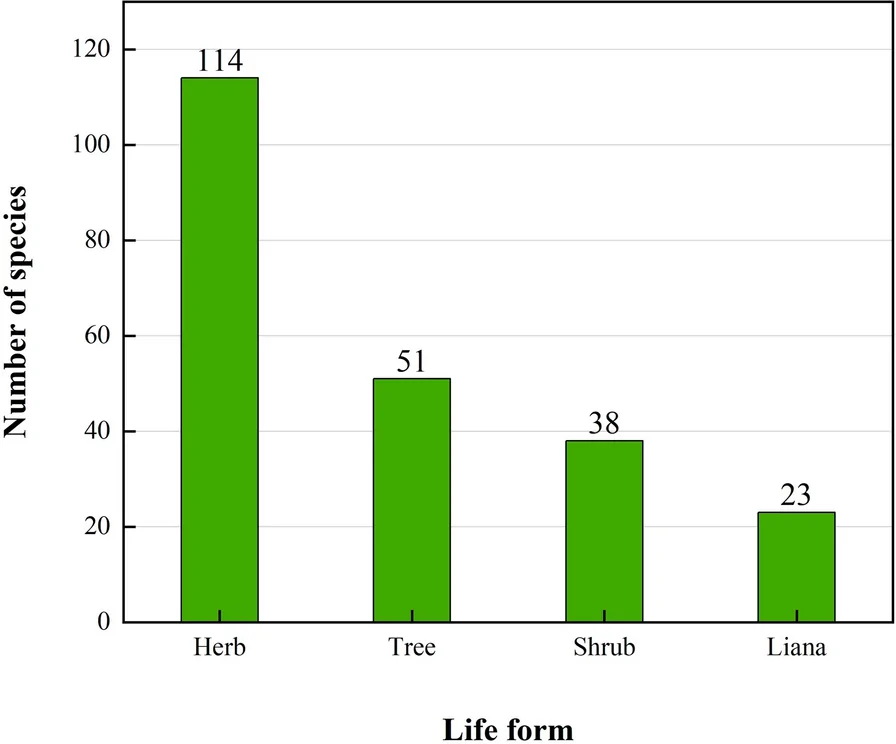
Life forms of medicinal plants

In terms of life form, medicinal plants in the study area were dominated by herbs, with 114 species accounting for 50.44% of the total. These were followed by trees, with 51 species (22.57%), shrubs, with 38 species (16.81%), and lianas, with 23 species (10.18%) (Fig. 4). Although medicinal plants of different life forms were all represented to a certain extent, herbaceous plants far outnumbered trees, which is largely consistent with the findings of related studies conducted both in China and abroad [37, 38]. The widespread use of herbaceous plants may be mainly attributed to the Jinuo people’s preference for using freshly collected medicinal materials obtained nearby and their traditional practice of collecting and using them immediately. In addition, herbs are easier to collect than woody plants such as trees and shrubs [39], and they generally have shorter growth cycles. Moreover, severe forest destruction in surrounding areas, which has resulted in a decline in woody plant resources, is also a factor that cannot be ignored [40].

**Fig. 5:**
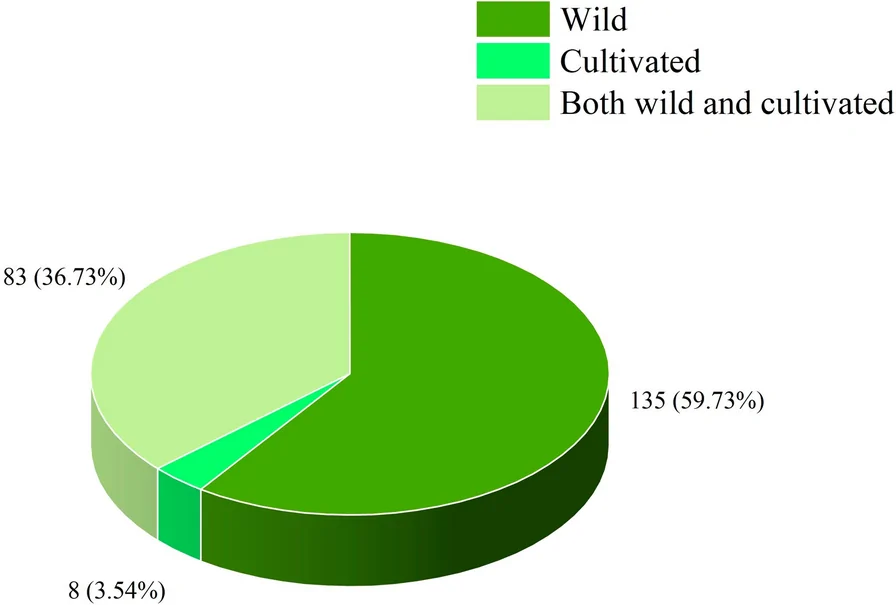
Habitats of medicinal plants

In terms of habitat, among the 226 medicinal plant species recorded in the study area, most species (135, 59.73%) were collected from wild habitats, while 83 species (36.73%) were obtained from both home gardens and wild habitats. Only 8 species (3.54%) were cultivated exclusively in home gardens (Fig. 5). Most medicinal plants were collected directly from the natural environment by local traditional Jinuo healers, whereas some introduced species or plants that were difficult to obtain locally were purchased from medicinal material suppliers. Fresh medicinal materials, in particular, were usually gathered from the wild [11].

### Traditional uses and preparation of medicinal plants

**Fig. 6:**
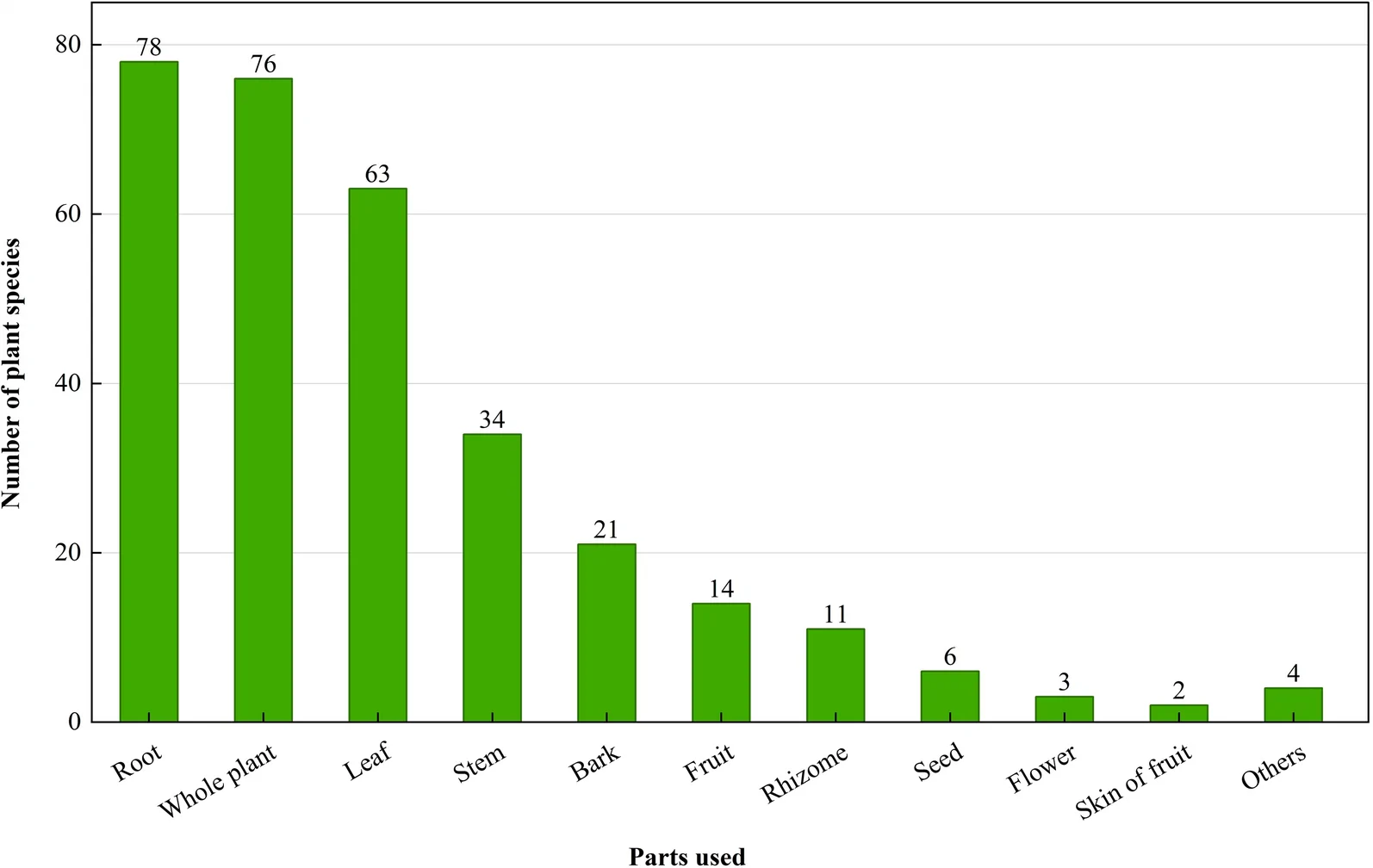
Plant parts used

Informants in the study area used different plant parts in the preparation of traditional medicines, mainly including roots, whole plants, leaves, stems, bark, fruits, rhizomes, and seeds (Fig. 6). A total of 13 different plant parts were recorded as being used for medicinal preparation. Roots were the most frequently used plant part, involving 78 species, followed by whole plants (76 species), leaves (63 species), stems (34 species), bark (21 species), fruits (14 species), rhizomes (11 species), seeds (6 species), flowers (3 species), peels (2 species), and other parts (such as heartwood, tubers, and kernels) from 4 species.

The medicinal parts of plants are closely related to their therapeutic effects, as different parts of the same plant may have distinct uses and functions [41, 42]. Through long-term practice, the Jinuo people have learned to use different parts of the same plant to treat different ailments according to their modes of use and medicinal effects [23]. Accordingly, when counting the medicinal parts used by the Jinuo people, repeated records occurred because different parts of the same plant were used medicinally. In total, 312 use-reports of medicinal plant parts were recorded. In terms of medicinal processing, traditional Jinuo healers primarily use fresh plant materials, following a practice of harvesting and using them immediately. Dried materials are generally limited to plants that are difficult to obtain or to annual herbs collected during their peak growing season and dried for later use [43]. The use of whole plants as medicine is common in many traditional medical systems, as healers often believe that this practice enhances efficacy [44]. Although such harvesting practices may cause some damage to local medicinal plant resources and may affect biodiversity, it is worth noting that some villagers have begun cultivating commonly used medicinal plants in their home gardens as alternative sources. In addition, in recent years, the county government has taken measures such as afforestation and related management practices to protect medicinal plant resources [45]. As a result, the problems associated with destructive harvesting and digging have been alleviated to some extent [41].

Based on statistical analysis of the medicinal plant records collected during field interviews, decoction for oral administration and external application of pounded plant materials were identified as the two principal methods of preparation and administration. Among these, decoction was the most common method, involving 185 species. In addition, 69 species were pounded and applied externally, mainly for the treatment of fractures, traumatic injuries, and snake or insect bites. Another 16 species were boiled in water and used to wash affected areas, mainly for the treatment of skin diseases. Other modes of use included soaking in alcohol for oral consumption, mixing with vinegar for topical application, eating raw, and stewing with meat. Stewing medicinal plants with chicken or pork is believed to provide nutritional supplementation and enhance medicinal efficacy [46]. Overall, medicinal plants were mostly collected locally and used fresh, and their preparation methods and dosage forms were relatively simple. Most recorded medicinal plants were prepared as decoctions, a method considered one of the most effective for extracting beneficial secondary metabolites [47, 48].

### Diseases treated in the study area

In the study area, these medicinal plants were used to treat 94 human diseases (Table 5). All diseases were identified and classified according to the International Classification of Diseases, 11th Revision (ICD-11), based on the healers’ descriptions and interpretations, and were ultimately grouped into 13 disease categories. Among these categories, traumatic diseases accounted for the highest number of medicinal plant species, with 105 species (17.13%), mainly used for bruises and sprains (54 species), fractures (25 species), venomous snake and insect bites (12 species), traumatic bleeding (9 species), and burns and scalds (5 species). Respiratory diseases ranked second, with 103 medicinal plant species (16.80%) used to treat 11 specific diseases, followed by digestive system diseases, with 95 species (15.50%) used to treat 14 specific diseases.

**Table 5:** Diseases treated in the study area

Disease category	Specific diseases (number of plant species used for treatment)
Diseases of the respiratory system	Common cold (31), fever (11), bronchitis (13), tracheitis (8), tonsillitis (11), asthma (1), pneumonia (4), emphysema (2), respiratory tract infection (2), pharyngolaryngitis (7), cough (13)
Diseases of the digestive system	Stomachache (16), abdominal pain (8), gastric ulcer (4), indigestion (16), gastroenteritis (6), cholecystitis (1), enteritis (16), diarrhea (16), gallstones (3), appendicitis (1), constipation (1), hernia (3), hemorrhoids (1), anal prolapse (3)
Diseases of the circulatory system	Hypertension (2), heart disease (2), pulmonary heart disease (1), lymphadenitis (1)
Diseases of the genitourinary system	Nephritis (14), urethritis (3), cystitis (6), kidney stones (4), urinary calculi (2), urinary tract infection (5)
Diseases of the nervous system	Neurasthenia (4), epilepsy (1), pediatric meningitis (1), febrile convulsions (1), headache (11)
Diseases of the musculoskeletal system	Sciatica (1), rheumatic diseases (7), rheumatoid arthritis (16), rheumatic pain (21), chronic osteomyelitis (1), bone hyperplasia (2), arthritis (3)
Gynecological diseases	Irregular menstruation (9), menorrhagia (2), amenorrhea (5), dysmenorrhea (2), uterine prolapse (1), mastitis (5), postpartum abdominal pain due to blood stasis (2), excessive vaginal discharge (1), postpartum weakness (2), abnormal uterine or vaginal bleeding (6)
Otorhinolaryngological and ophthalmic diseases	Eye diseases (4), otitis media (1), toothache (7), stomatitis (4)
Diseases of the skin	Allergy (4), urticaria (1), eczema (9), abscess (2), pruritus (5), tinea (2), dermatitis (3), furuncle (10), carbuncle (4), tinea pedis (1), scabies (1), skin and soft tissue infection (4)
Traumatic injuries	Traumatic bleeding (9), sprains and contusions (54), fractures (25), burns and scalds (5), bites from venomous snakes and poisonous insects (12)
Diseases of the endocrine system	Diabetes mellitus (1), endemic goiter (1)
Infectious diseases	Pulmonary tuberculosis (7), measles (2), hepatitis (28), mumps (4), malaria (18), influenza (1), pertussis (3), gonorrhea (1), dysentery (28)
Other diseases	Anemia (2), lymphatic tuberculosis (3), heat stroke (2), tumor (1), impotence (2)

**Fig. 7:**
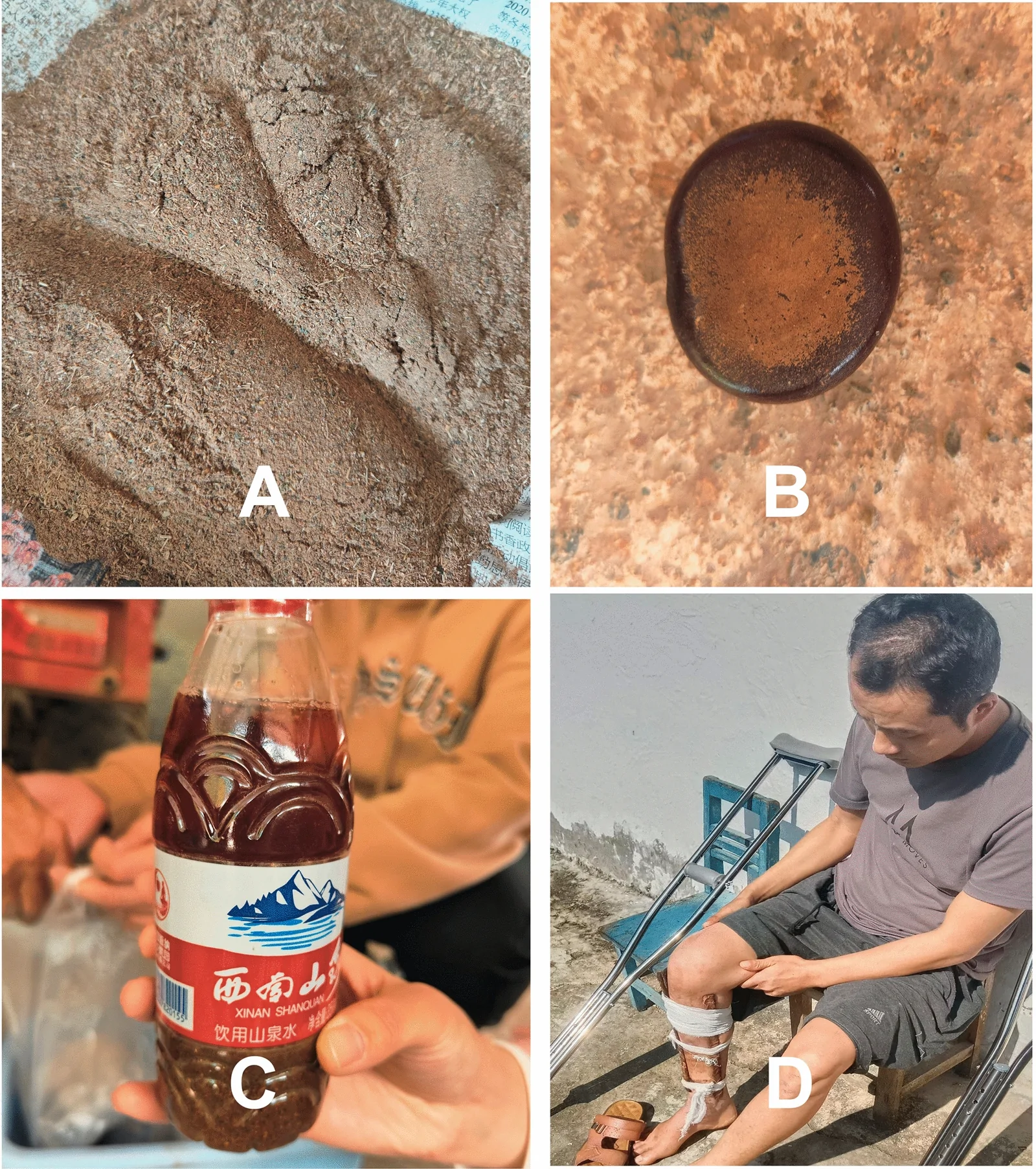
Secret remedies for treating traumatic injuries and bone fractures. **A** Specially prepared herbal powder, **B** Fruit of the vine, **C** Homemade medicinal wine, **D** Fracture treatment

**Fig. 8:**
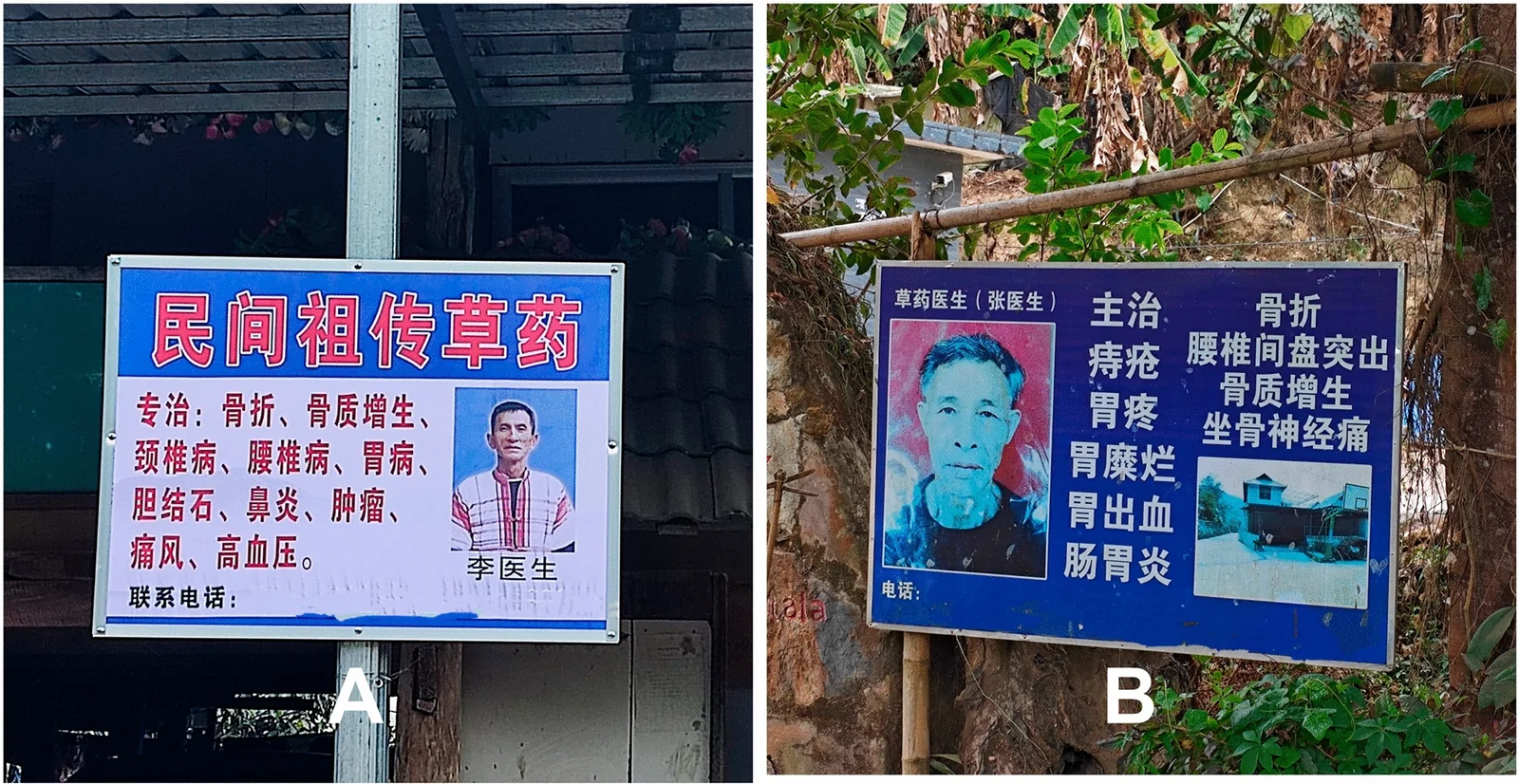
Signboards for traditional Jinuo healers. **A** Mr. Li, specializing in bone fractures, stomach diseases, gallstones, and more. **B** Mr. Zhang, specializing in bone fractures, bone spurs, stomach diseases, and more

The results showed that Jinuo traditional medicine is mainly used to treat digestive diseases, respiratory diseases, and traumatic injuries. This pattern may be related to the local natural environment, dietary structure, and modes of production and daily life. Xishuangbanna has a mean annual temperature of 18–20 °C and an annual precipitation of approximately 1100 mm, and is characterized by a north tropical southwest monsoon climate [49]. Temperature and humidity are important meteorological factors influencing the occurrence of digestive and respiratory diseases. Higher temperature and humidity favor the growth and reproduction of bacteria and other pathogenic microorganisms, thereby increasing the risk of respiratory and gastrointestinal diseases [50]. In addition, the Jinuo people prefer glutinous rice, hot and sour foods, and cold dishes, and have retained the tradition of consuming raw meat products, such as “duosheng”, which may to some extent increase the risk of gastrointestinal discomfort and digestive diseases [31]. Moreover, local residents have long been engaged in agricultural and forestry work, and traumatic injuries such as fractures, bruises, and sprains are relatively common. Therefore, the treatment of such injuries has also become an important field of Jinuo traditional medicine. Field investigations showed that many traditional Jinuo healers are skilled in treating fractures, bruises, and sprains, and preserve family-inherited secret remedies (Fig. 7). Some healers also display signboards in front of their houses and along roadsides (Fig. 8).

### Medicinal and edible plants used by the Jinuo people

Among the 226 medicinal plant species recorded in the study area for the treatment of human diseases, some were also reported to have edible value. Of these, 40 commonly used medicinal and edible plant species were identified in local communities, and their plant names, edible parts, edible uses, medicinal parts, and medicinal uses are listed in Table 6. The Jinuo people have developed diverse ways of utilizing these plants. Common methods of use include eating raw, serving cold, stewing, stir-frying, soaking in water, soaking in liquor, decocting in water, and pounding.

**Table 6:** Commonly used medicinal and edible plants in the study area

No	Scientific name & Chinese name	Edible parts	Edible uses	Medicinal parts	Medicinal uses
1	*Plantago asiatica* L 车前	Leaf	Eaten in salads; stir-fried; soaked in water and drunk as an infusion; boiled with brown sugar	Whole plant	Decoction taken orally
2	*Gynostemma pentaphyllum* (Thunb.) Makino绞股蓝	Leaf	Eaten in salads; stir-fried; used in soups; soaked in water and drunk as an infusion	Whole plant	Decoction taken orally; applied externally as a poultice
3	*Psidium guajava* L 番石榴	Fruit, young leaf	Fruits eaten raw; young leaves soaked in water as an infusion or boiled with brown sugar	Leaf, fruit	Decoction taken orally; chewed fresh; pounded and applied externally
4	*Phyllanthus emblica* L.余甘子	Fruit, bark	Fruits eaten raw or soaked in liquor; bark chopped and eaten raw	Root, leaf, bark	Decoction taken orally; boiled in water and used for external washing
5	*Spondias pinnata* (L. F.) Kurz槟榔青	Fruit, young leaf	Fruits eaten raw; young leaves eaten in salads or stir-fried after blanching	Bark, fruit	Decoction taken orally
6	*Fagopyrum dibotrys* (D. Don) Hara金荞麦	Whole plant	Used in soups; eaten in salads; cooked with eggs	Rhizome, leaf	Decoction taken orally; soaked in wine and taken orally; pounded and applied externally
7	*Houttuynia cordata* Thunb.蕺菜	Rhizome, leaf	Eaten in salads; stir-fried; used in soups or as a seasoning vegetable; roots soaked in water with honey	Whole plant	Decoction taken orally
8	*Centella asiatica* (L.) Urb.积雪草	Whole plant	Eaten in salads; pickled; used in soups; stir-fried; soaked in water and drunk as an infusion	Whole plant	Decoction taken orally
9	*Bombax ceiba* L.木棉	Flower, fruit	Dried and then used in soups, porridge, or tea	Bark, root	Pounded and applied externally
10	*Strobilanthes cusia* (Nees) Kuntze板蓝	Young stem and leaf	Stir-fried; eaten in salads; used in soups	Root, leaf	Pounded and applied externally; decoction taken orally
11	*Pueraria montana* var. *lobata* 葛	Young stem and leaf, flower, root	Young leaves eaten in salads, stir-fried, or used in soups after blanching; flowers dried and used as tea; roots eaten raw or soaked in water as an infusion	Root	Decoction taken orally
12	*Oroxylum indicum* (L.) Kurz木蝴蝶	Flower, young fruit pod, young stem and leaf	Stir-fried; cooked in banana leaves; eaten after blanching with nammi dipping sauce	Seed, bark	Soaked in water and taken orally
13	*Solanum nigrum* L 龙葵	Leaf	Young leaves eaten in salads; used in soups; stir-fried after blanching	Whole plant	Decoction taken orally; pounded and applied externally
14	*Cymbopogon citratus* (DC.) Stapf柠檬草	Whole plant	Used as a seasoning for grilled fish and for soups	Whole plant	Decoction taken orally
15	*Artemisia argyi* H. Lév. & Vaniot艾	Young leaf	Pounded into a paste and mixed with glutinous rice flour to make various pastries	Whole plant	Boiled in water and used for external washing
16	*Solanum spirale* Roxb.旋花茄	Leaf, fruit	Eaten in salads; stir-fried; used in soups	Root	Decoction taken orally
17	*Zingiber officinale* Roscoe姜	Rhizome	Eaten in salads; stir-fried; pickled; also used to make ginger tea or as a seasoning	Rhizome	Decoction taken orally
18	*Amorphophallus konjac* K. Koch魔芋	Rhizome	Repeatedly boiled and washed before eating	Rhizome	Decoction taken orally; heated and applied to the affected area
19	*Oenanthe javanica* (Blume) DC.水芹	Whole plant	Eaten raw; boiled; stir-fried	Whole plant	Decoction taken orally; may also be eaten raw
20	*Zanthoxylum armatum* DC.竹叶花椒	Young leaf, skin of fruit, seed	Used in soups; made into tea; used as a spice	Root, leaf	Decoction taken orally; pounded and applied externally
21	*Caryota maxima* Blume ex Mart.鱼尾葵	Fruit	Eaten after cooking; used for brewing liquor	Root, leaf	Decoction taken orally; pounded and applied externally
22	*Aralia decaisneana* Hance台湾毛楤木	Young shoot and young stem	Eaten in salads; stir-fried; used in soups	Root	Decoction taken orally
23	*Polygonatum cirrhifolium* (Wall.) Royle卷叶黄精	Root, stem	Soaked in water as an infusion; used in soups	Root, stem	Decoction taken orally
24	*Areca catechu* L.槟榔	Kernel	Chewed and eaten raw	Fruit	Decoction taken orally
25	*Bidens pilosa* L.鬼针草	Leaf, stem	Eaten in salads; stir-fried; used in soups	Whole plant	Decoction taken orally; pounded and applied externally
26	*Tamarindus indica* L 酸豆	Fruit	Eaten raw; pickled; eaten in salads; used to season soups and stews	Bark	Decoction taken orally
27	*Ficus semicordata* Buch.-Ham. ex Sm.鸡嗉子榕	Fruit, leaf	Fruits eaten raw; young leaves eaten in salads or stir-fried	Skin of fruit	Decoction taken orally
28	*Rubus ellipticus* var. *obcordatus* (Franch.) Focke栽秧藨	Fruit	Eaten raw	Root	Decoction taken orally
29	*Eryngium foetidum* L 刺芹	Young stem and leaf	Used for cooking fish in banana leaves; used in soups; eaten in salads; stir-fried	Whole plant	Decoction taken orally
30	*Musa acuminata* Colla小果野蕉	Young leaf, inflorescence	Eaten in salads; used in soups; stir-fried with cured meat; cooked in banana leaves	Fruit, flower	Pounded and applied externally
31	*Elaeagnus conferta* Roxb.密花胡颓子	Fruit	Eaten raw; soaked in water and drunk as an infusion	Root, fruit	Decoction taken orally
32	*Rorippa indica* (L.) Hiern蔊菜	Young stem and leaf	Eaten in salads; stir-fried; used in soups; stir-fried with meat	Whole plant	Decoction taken orally
33	*Lablab purpureus* (L.) Sweet扁豆	Young pod	Stir-fried; stewed with meat	Leaf	Decoction taken orally
34	*Melastoma malabathricum* L.印度野牡丹	Fruit	Eaten raw; soaked in water and drunk as an infusion	Whole plant	Decoction taken orally
35	*Ficus auriculata* Lour.大果榕	Young leaf	Stir-fried; used in soups; eaten in salads	Leaf	Decoction taken orally
36	*Solanum torvum* Sw 水茄	Fruit	Used in soups; stewed with meat; pickled; dried for use in medicinal cuisine	Fruit	Decoction taken orally; pounded and applied externally
37	*Crateva unilocularis* Buch.-Ham.树头菜	Leaf	Eaten after pickling; stir-fried; used in soups; eaten in salads	Bark, leaf	Decoction taken orally
38	*Embelia ribes* Burm. F.白花酸藤果	Leaf, fruit	Eaten raw or in salads	Root, leaf, stem	Decoction taken orally
39	*Pteridium revolutum* (Blume) Nakai毛轴蕨	Young leaf, young stem	Eaten in salads; stir-fried	Root	Decoction taken orally
40	*Litsea pungens* Hemsl.木姜子	Fruit	Stewed with chicken; cooked with fish; used as a seasoning	Root	Decoction taken orally

Due to the hot and humid climate of the Jinuo Mountain region, the dietary choices and taste preferences of the local residents are largely influenced by this natural environment. Under these conditions, raw and cold foods are common, bitter foods have a cooling effect, sour foods are refreshing and appetizing, and spicy dishes help to dispel wind and dampness [36]. Therefore, local residents often consume certain fruits and vegetables raw or as cold dishes seasoned with spices, including some species with both edible and medicinal value. For example, the young leaves of *Psidium guajava* and the whole plant of *Oenanthe javanica* can be eaten raw, while the slightly bitter leaves of *Solanum nigrum* and *Solanum spirale* are commonly stir-fried. The whole plant of *Fagopyrum dibotrys* is often served cold with seasonings such as salt and chili. In addition, the bark of *Phyllanthus emblica* and the fruits of *Litsea pungens* are sometimes used as traditional seasonings for raw minced meat dishes. Some medicinal plants are also prepared as herbal teas for daily healthcare. For instance, *Plantago asiatica* and *Gynostemma pentaphyllum* are commonly consumed by soaking or boiling them in water and are believed to have heat-clearing and detoxifying effects. The Jinuo people also have a tradition of consuming flowers, and the inflorescences of *Musa acuminata* are commonly eaten after stir-frying, boiling, steaming, or wrapped roasting [51].

In addition to the common modes of consumption described above, locally distinctive processing and combined-use practices have also been developed for some medicinal and edible plants within the Jinuo community. *Amorphophallus konjac* provides a representative example. Its leaves and petioles can be consumed as wild vegetables. However, when stir-fried alone, they often produce a pronounced acrid or numbing taste, which affects their palatability. Through long-term dietary practice, local residents have developed a method of cooking this plant together with sour ingredients. It is commonly stir-fried with pickled vegetables or sour papaya to reduce the numbing taste and improve its flavor. This practice reflects the Jinuo people’s empirical knowledge of plant taste, irritant properties, and food combinations, and indicates that a relatively stable body of knowledge concerning plant processing and combined use has been formed in their daily diet.

### Ethnobotanically important medicinal plants based on RFC values

**Fig. 9:**
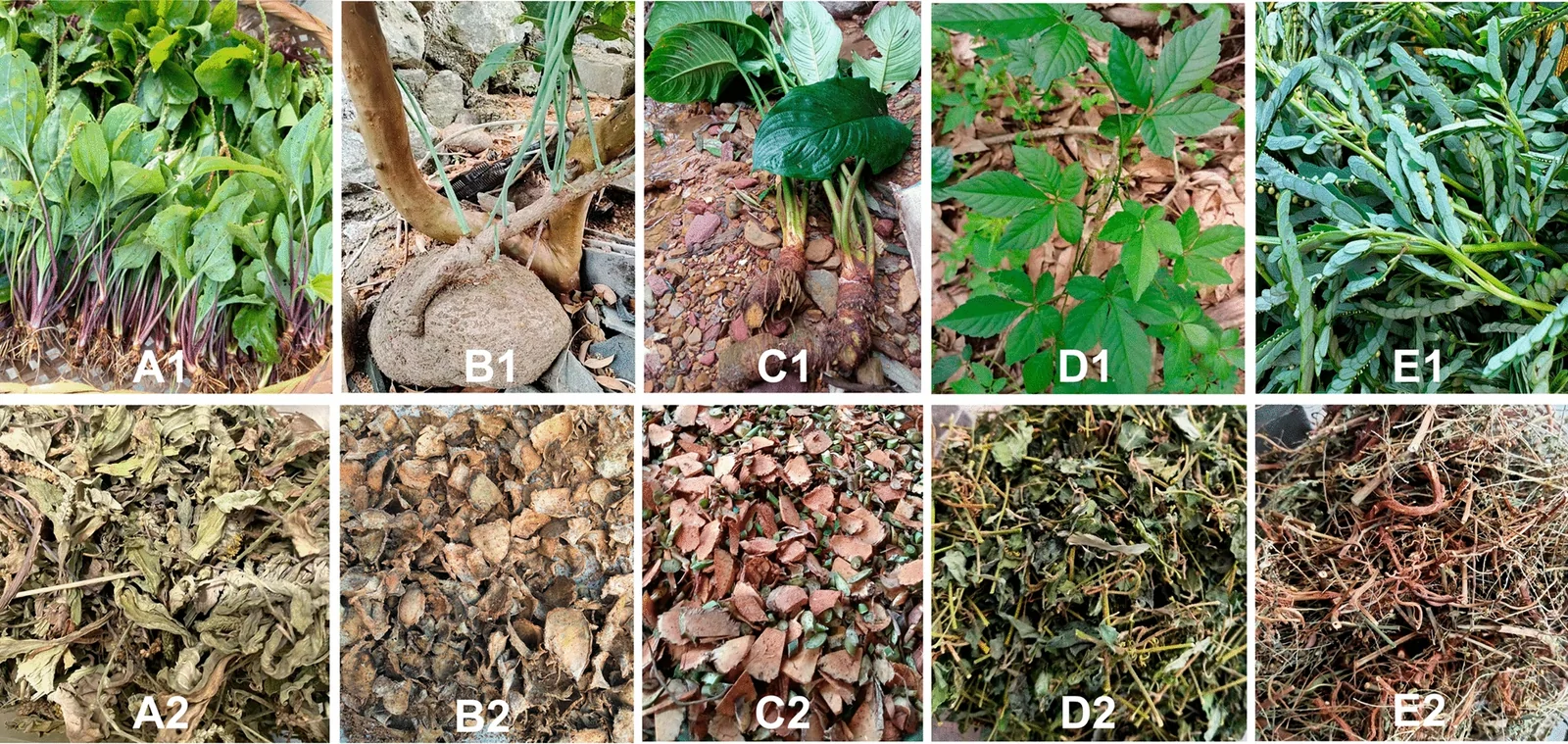
Fresh and dried medicinal plants. **A** *Plantago asiatica* L., **B** *Stephania epigaea* H. S. Lo, **C** *Tacca chantrieri* André, **D** *Gynostemma pentaphyllum* (Thunb.) Makino, **E** *Phyllanthus urinaria* L. Here, 1 indicates the fresh material and 2 indicates the dried material

The RFC values of medicinal plants were calculated to reflect their local importance. The results showed that *Plantago asiatica* (0.87), *Stephania epigaea* (0.86), *Tacca chantrieri* (0.85), *Gynostemma pentaphyllum* (0.85), and *Phyllanthus urinaria* (0.84) had relatively high RFC values (Fig. 9), suggesting that these species are frequently used by traditional Jinuo healers and are also well known among local villagers. Among them, *Plantago asiatica* is widely distributed in the Jinuo Mountains, mainly growing along roadsides, riverbanks, and open areas near villages. Owing to its medicinal and edible value, it is widely recognized by local people, who refer to it as “cool medicine,” a local term for medicinal plants believed to clear internal heat, and often consume it as a vegetable. Its leaves are also infused in water or boiled with brown sugar and consumed as a drink. The whole plant is usually harvested in summer and autumn and used either fresh or after drying. It is mainly used to treat fever, dysuria, and urinary calculi. In ethnic minority regions of Guizhou, *Plantago asiatica* is used to enhance immunity and reduce blood uric acid levels [52]. Recent pharmacological studies have indicated that tannins, the major active compounds in this plant, may alleviate diarrhea by binding to proteins and reducing intestinal secretions [53].

*Stephania epigae* is an important medicinal plant used by the Jinuo people for the treatment of fractures, bruises, and sprains, and it is also used to treat abdominal pain, stomachache, and metrorrhagia. It can be harvested throughout the year. In most cases, the fresh material is used immediately after collection, or the tuber is sliced and dried for later use. Recent studies have shown that the tuber of this species contains relatively high amounts of cepharanthine and cycleanine, both of which have been applied clinically and have achieved good therapeutic effects [54]. Cepharanthine, as a drug for the prevention and treatment of leukopenia, has shown good clinical efficacy and is mainly used to prevent and treat leukopenia caused by radiotherapy and chemotherapy in cancer patients [55]. In addition, cepharanthine also exhibits notable antiviral activity [56, 57]. Moreover, iodinated derivatives of cycleanine have muscle relaxant effects and can effectively prevent and treat experimental silicosis in animals [54]. Overall, *Stephania epigaea* has great medicinal and economic value and deserves further development.

*Tacca chantrieri* is a perennial herb that mainly grows in shady and humid habitats in tropical forests and has high medicinal and scientific value. Its medicinal parts are mainly the rhizomes and leaves, which can be used both internally and externally. The rhizomes are decocted for the treatment of pharyngitis, pneumonia, malaria, and gastric disorders, whereas the crushed leaves are applied externally to relieve traumatic bruising and swelling. It also has high ornamental value because of its attractive plant form, distinctive flowers, and long flowering period. However, wild populations of *Tacca chantrieri* have gradually declined and show an endangered trend [58]. Its seed germination requires both sufficient moisture and light, which are difficult to obtain simultaneously in the tropical rainforest understory, resulting in poor natural reproduction. Meanwhile, habitat destruction caused by human activities, such as deforestation and land reclamation, has further reduced its population size. As a result, this species is now rarely found near villages, and collection often requires travel to remote rainforest areas far from human settlements.

*Gynostemma pentaphyllum* is a medicinal and edible plant. The whole plant can be used medicinally and processed into beverages or herbal tea. Its young stems and leaves are also consumed as a vegetable. Because of the hot and humid climate in Jinuo inhabited areas, local people often use this species to treat colds, fever, sore throat, and bronchitis. Traditional Jinuo knowledge also holds that it has beneficial effects in reducing blood pressure and blood lipids, which has been supported by modern studies [59]. Previously, *Gynostemma pentaphyllum* was mainly collected from the wild, but many Jinuo villagers now cultivate it under forest canopies or along field margins. The harvested material is sold for further processing into tea bags and dried products, which are marketed as local specialty products or tourist commodities and provide an additional source of income for local communities.

*Phyllanthus urinaria* is one of the tea and medicinal plants most frequently used in Jinuo communities and plays an important role in local traditional medicine and daily healthcare. The whole plant is commonly used as a tea substitute and can be collected throughout the year. Local residents usually boil the fresh or sun-dried whole plant in water for about 20 min and drink the decoction as tea. The infusion is light green, aromatic, slightly bitter, and followed by a sweet aftertaste, and is believed to clear heat, promote diuresis, improve eyesight, and relieve food retention. It is commonly consumed as a daily health drink to prevent or relieve urinary tract infections, enteritis, dysentery, and hepatitis. *Phyllanthus urinaria* is also used externally in folk practice. Fresh plants are crushed and applied to the affected area to assist in the treatment of snakebites or relieve local inflammation. Because of its clear medicinal effects, easy accessibility, and simple preparation, this species is highly recognized and frequently used in the traditional plant-use system of the Jinuo people.

### Comparison of medicinal plants with other ethnic groups and previous Jinuo records

To compare the similarity of medicinal plants used by the Jinuo people with those used by other ethnic groups and regions, this study employed the Jaccard Similarity Index (JI). The results showed that the similarity in medicinal plant composition between the Jinuo people and other ethnic groups and regions was generally at a low to moderate level (Table 7).

**Table 7:** Jaccard similarity index for Jinuo people and other ethnic groups

Study area	Surveyed ethnic group	Number of species	Shared species	Jaccard similarity index	References
Jinuo Mountain Jinuo Ethnic Township, Jinghong City, Xishuangbanna, Yunnan	Jinuo people	226	-	-	Present study
Naban River Watershed National Nature Reserve, Xishuangbanna, Yunnan	Hani people	199	57	15.49	[60]
Xishuangbanna, Yunnan	Dai people	226	58	14.72	[61]
Huanjiang Maonan Autonomous County, Guangxi	Maonan people	368	69	13.14	[11]
Menghai County, Xishuangbanna, Yunnan	Bulang people	60	28	10.85	[8]
Miao settlements in Hainan Province	Miao people	348	55	10.60	[62]
Sandu Shui Autonomous County, Guizhou	Shui people	505	56	8.30	[41]
Kaili markets, Qiandongnan Miao and Dong Autonomous Prefecture, Guizhou	Miao and Dong people, and other ethnic groups	237	34	7.93	[63]
Xiaoliangshan Area, Ninglang Yi Autonomous County, Yunnan	Yi people	125	15	4.46	[43]

The highest similarity was observed between the Jinuo people and the Hani people in the Naban River Watershed (JI = 15.49), with 57 medicinal plant species shared between them. Both areas are located in Xishuangbanna and are geographically close to each other, with similar elevational ranges and vegetation types. These comparable ecological conditions, together with a long-term reliance on forest plant resources, may contribute to the relatively higher overlap in medicinal plant use. However, even within Xishuangbanna, the number of shared species remained limited, suggesting that local selection and use of medicinal plants are not determined solely by ecological availability, but are also closely associated with ethnic culture, disease perception, and modes of knowledge transmission. The Jinuo medicinal plants also showed relatively high similarity with the medicinal and edible plants commonly used by the Dai people in Xishuangbanna (JI = 14.72), with 58 shared plant species. The Dai people generally inhabit basin areas or river-valley plains, whereas the Jinuo people mainly live in mountainous areas such as Jinuo Mountain. Therefore, the two groups differ in their residential environments and in the spaces from which they commonly obtain plant resources. Nevertheless, both groups live in Xishuangbanna, where regional plant resources partly overlap. In addition, Dai medicinal and edible plants are closely associated with daily diets, disease prevention, and household healthcare. Some commonly used plants may therefore be recognized and used by different ethnic groups through farmers’ markets, medicinal plant markets, village-level interactions, and other forms of local exchange. These factors may explain the relatively high similarity in plant use between the Jinuo and Dai people in Xishuangbanna.

The largest number of shared medicinal plant species was recorded between the Jinuo people and the Maonan people in Guangxi province, with 69 shared species and a JI value of 13.14. The Maonan area is characterized by a subtropical humid mountainous environment, which shows some similarities to Jinuo Mountain in terms of climate and montane vegetation. However, the karst landform of the Maonan area differs from the tropical montane rainforest of Xishuangbanna. In addition, the Maonan study recorded a larger total number of medicinal plant species, which may partly explain why its similarity with the Jinuo people was lower than that observed for the Hani and Dai groups in Xishuangbanna.

Moderate similarity values were recorded between the medicinal plants of the Jinuo people and those of the Bulang people in Menghai and the Miao people in Hainan, with JI values of 10.85 and 10.60, respectively. The Bulang and Jinuo people are both mountainous ethnic groups in southern Yunnan. However, the higher elevation of the Bulang people's settlement may limit further overlap in medicinal plant composition. The areas inhabited by the Hainan Miao people are also characterized by a tropical monsoon climate and rich plant resources, resulting in a certain number of medicinal plant species shared with the Jinuo people. However, the long geographical distance between Hainan and Xishuangbanna, together with differences in ethnic cultural backgrounds and plant-use practices, may restrict further overlap in medicinal plant composition, keeping the overall similarity at a moderate level.

In contrast, lower similarity values were recorded between the Jinuo people and the Shui people, the Kaili medicinal plant market, and the Yi people, with JI values of 8.30, 7.93, and 4.46, respectively. The lowest similarity was observed between the Jinuo people and the Yi people in Xiaoliangshan, with only 15 medicinal plant species shared between them. This may be related to the higher elevation, lower mean annual temperature, and plateau mountainous environment of Xiaoliangshan, which differ markedly from the tropical rainforest environment of Jinuo Mountain.

The similarity in medicinal plant composition between the Jinuo people and other ethnic groups and regions showed a clear pattern of ecogeographical differentiation. Studies with relatively higher similarity values were mostly associated with geographical proximity, comparable climatic conditions, or similar mountainous forest environments to the Jinuo study area, and may also have been influenced by regional plant-use experience and knowledge exchange among ethnic groups. In contrast, studies with lower similarity values were generally located in areas with more distinct ecological environments. In the comparison between the present study and the eight previous ethnobotanical studies, only *Plantago asiatica* was recorded as medicinal plants in all studies. *Plantago asiatica* had the highest RFC value in the Jinuo communities, indicating its high level of recognition and frequency of use locally. It is also an important medicinal resource commonly used in the traditional medical systems of several ethnic groups. Overall, medicinal plant knowledge among the Jinuo people still shows clear local specificity and ecological adaptability.

In addition to comparisons with medicinal plant records from other ethnic groups and regions, the present study was also compared with existing records of Jinuo medicinal plants to assess newly documented information and local variation [21, 23, 24]. Most of the 226 medicinal plant species recorded in this study had been reported previously, and no large number of new species records was found. Differences between this study and previous records were mainly related to medicinal parts, including additions, omissions, or more detailed descriptions of plant parts used. Substantial differences in medicinal uses were found for only five species, namely *Cryptolepis buchananii*, *Mallotus barbatus*, *Adenanthera microsperma*, *Musa acuminata*, and *Curcuma phaeocaulis*. These differences suggest that Jinuo medicinal plant knowledge largely continues the existing knowledge system, while showing some local variation. It should be noted that differences in recorded medicinal parts may be influenced by informant selection, recording detail, terminology, and changes in current community practice. Therefore, such differences are regarded here as variation in traditional knowledge documentation rather than new discoveries of medicinal plants.

## Discussion

### The role of medicinal plants and traditional knowledge in biodiversity conservation

Medicinal plants and their associated traditional knowledge play an important role in biodiversity conservation. The sustainable management of medicinal plant resources contributes to the sustainable development of both forest ecosystems and rural economies. The value of forests lies not only in timber production, but also in the ecological and economic benefits generated by diverse non-timber forest products [64, 65]. As an important component of non-timber forest products, medicinal plants can, through appropriate harvesting and management, generate economic returns comparable to those of alternative land-use systems such as intensive agriculture or plantation forestry, while also providing an important source of household income for forest-dependent communities [66, 67]. Moreover, medicinal plants serve as important resources and model references for new drug development [68]. Therefore, the sustainable use of medicinal plant resources is important not only for biodiversity conservation, but also for providing a material basis for the development of traditional medicine.

However, the conservation of medicinal plants is not limited to the protection of plant resources themselves, but is also closely associated with the traditional knowledge that supports their utilization and management. In many parts of the world, traditional knowledge and biodiversity continue to play important roles in healthcare, culture, religion, food security, environmental protection, and sustainable development. Moreover, many widely used plant-derived medicines originate from traditional knowledge [69]. Therefore, the documentation, conservation, and, where supported by scientific evidence, promotion of traditional knowledge are of great significance. The Global Strategy for Plant Conservation (2020–2030) also emphasizes that, with free, prior, and informed consent, the long-accumulated knowledge and experience of Indigenous peoples and local communities in plant use and management should be incorporated into plant biodiversity conservation actions so as to promote ecological restoration, agrobiodiversity conservation, and the sustainable use of plant resources [70]. This indicates that traditional knowledge is not merely a form of cultural heritage, but also an important knowledge resource that can contribute to modern biodiversity conservation practices.

In the Jinuo region, traditional knowledge does not exist in an abstract form, but participates in the conservation of medicinal plant resources and their habitats through customary laws, village regulations, religious rituals, and daily resource management practices. Through the long term use and management of natural resources, the Jinuo people have developed a series of behavioral norms for protecting water sources, land, and forest resources [71]. To some extent, these traditional norms have restricted the overexploitation of forest resources and maintained the habitats on which medicinal plants depend. In traditional agricultural rituals such as the Dawopeng ritual, which is closely associated with shifting cultivation practices, village elders plant medicinal species such as *Zingiber officinale* and *Amorphophallus konjac* in front of old ritual huts. Such cultural practices not only have symbolic and religious significance, but also indirectly promote the conservation of local wild medicinal plant germplasm resources. These traditional management systems and cultural practices have enabled the ecosystems surrounding Jinuo villages to remain in relatively good condition over a long period, demonstrating the practical value of traditional knowledge in medicinal plant conservation and biodiversity maintenance.

Comparisons across cultures and regions further indicate that the conservation value of medicinal plants and their associated traditional knowledge depends not only on plant resources themselves, but also on the cultural knowledge systems that support plant use. The relatively high similarity between the Jinuo people and neighboring ethnic groups in Xishuangbanna, or groups inhabiting similar humid mountainous environments, suggests that geographical proximity and ecological similarity may have promoted the shared use of certain medicinal plants. However, the generally low to moderate levels of Jaccard similarity indicate that ecological factors alone cannot fully explain the formation of medicinal plant knowledge. Differences among ethnic groups in plant selection, medicinal preparation methods, and concepts of disease treatment may further reflect the influences of ethnic culture, dietary habits, local understandings of illness, and systems of knowledge transmission. These findings suggest that biodiversity conservation should not focus solely on plant species themselves, but should also pay attention to the local knowledge systems associated with plant use and inheritance. For medicinal plant resources of the Jinuo people, the conservation of plant diversity and the protection of traditional knowledge are mutually interdependent.

### Medicinal and edible plants as a link between diet and healthcare

Food-medicinal plants serve as an important medium linking the daily diet and household healthcare of the Jinuo people. The frequent use of these plants recorded in this study indicates that Jinuo medicinal plant knowledge is not limited to disease treatment, but is embedded in everyday life and dietary practices. This pattern is comparable to the concept of medicine-food homology, which recognizes a continuum between food and medicine [72]. Previous research suggests that some edible plants not only provide nutrients but are also used in daily practices for health maintenance and disease prevention [73]. In the humid mountainous environment of Jinuo Mountain, local preferences for raw, bitter, sour, and spicy foods reflect both the long-established dietary culture of the Jinuo people and their adaptation to the local climate and living environment [31]. In local understanding, bitter plants are often considered to have cooling effects, sour plants are believed to stimulate appetite, and spicy foods are regarded as helpful for dispelling dampness and wind. Therefore, medicinal and edible plants serve not only as nutritional resources, but also as important cultural resources through which the Jinuo people maintain health in daily life.

The medicinal and edible plants recorded in this study included wild vegetables, edible flowers, spice plants, herbal teas, and other types, indicating that local plant knowledge remains practically important for maintaining dietary diversity and household health. Maintaining these plant resources and their habitats is relevant not only to biodiversity conservation, but also to the ability of local communities to sustain their dietary traditions and health practices [74]. In this sense, the conservation of medicinal and edible plants can broadly support local food autonomy, that is, the ability of communities to maintain, use, and transmit their own food and health related practices [75].

From the perspective of environmental sustainability, medicinal and edible plants reflect the Jinuo people’s continuous utilization and knowledge of plant resources in surrounding forests, field margins, and village areas. These plants do not serve merely a single medicinal or dietary function. Rather, they fulfill multiple roles in daily life, including nutritional supplementation, disease prevention, home remedies, and cultural transmission [30]. Therefore, the conservation of medicinal and edible plants and their habitats can link biodiversity protection with the daily needs of local communities [76], facilitating comprehension and participation in conservation efforts. Moreover, species with high utilization frequency or multiple functions can be prioritized for future community resource monitoring, sustainable harvesting, and cultivation initiatives.

From a public health perspective, medicinal and edible plants still hold potential value in household healthcare and primary health care. Many of these plants are used for daily wellness or to treat common health issues such as digestive disorders, respiratory problems, skin diseases, rheumatism, and injuries [62, 77], providing relatively accessible health resources for rural and mountainous communities. However, if future efforts aim to integrate these local practices into public health initiatives, it is necessary to conduct pharmacological, toxicological, and safety evaluations while respecting the cultural context of the Jinuo people and the rights of knowledge holders, and to systematically document dosages, contraindications, preparation methods, and appropriate usage scenarios. Such research can help distinguish safe and effective local practices from potentially high-risk uses, and facilitate a rational integration of traditional knowledge into modern public health systems.

In addition, some plants possess cultural or spiritual significance in Jinuo traditional beliefs [78]. For example, *Zingiber officinale* is believed to ward off evil spirits and prevent misfortune [79]. Long-established dietary practices of the Jinuo people are closely associated with local knowledge of the identification, use, and management of wild medicinal plants. This knowledge system, which integrates food and medicine, supports the continued use and transmission of knowledge concerning valuable plant resources. Overall, food-medicinal plants link diet, household healthcare, and cultural knowledge, highlighting the broader significance of Jinuo medicinal plant knowledge beyond their nutritional and therapeutic uses.

### Threats to medicinal plants and traditional knowledge

According to the survey results, villagers familiar with medicinal plants were mainly between 40 and 75 years old (72.30%), indicating that traditional knowledge of medicinal plants is largely held by older people. This finding is consistent with the results reported in several ethnobotanical studies [80, 81]. The survey further shows that the transmission of medicinal plants and associated traditional knowledge is currently threatened by multiple factors, most notably the declining number of traditional Jinuo healers and the difficulty of passing on this knowledge. Most traditional Jinuo healers have neither undergone official certification nor obtained licensed physician qualifications. As a result, their practice is relatively limited in scope, relying mainly on word of mouth, with patients coming mostly from nearby villages. Because the Jinuo people have no written language, traditional medical knowledge has long been transmitted orally. Young apprentices usually have to follow traditional Jinuo healers into the mountains to collect medicinal plants, learning through field practice to identify the plants and understand their medicinal uses, a process that is quite demanding. Meanwhile, the relatively low income from this practice also reduces young people’s willingness to pursue it as a profession. In addition, traditional medical knowledge is mostly passed down within families and often follows the custom of “passing to males but not to females.” Together with the strong secrecy surrounding folk prescriptions, this further limits the spread of knowledge and gradually narrows the foundation for the inheritance of traditional medical knowledge.

The availability of medicinal plant resources is also declining. In recent years, the expansion of cash crops such as rubber and tea has significantly changed land-use patterns [82]. Some forests and native vegetation have been converted into economic plantations, leading to the continuous loss of medicinal plant habitats. At the same time, unregulated harvesting still occurs in some areas. Since many medicinal materials are obtained from roots, rhizomes, and whole plants, long-term overharvesting and habitat destruction have reduced some wild resources at the source, and certain species even face the risk of depletion. In addition, traditional knowledge related to the use of medicinal plants is gradually being lost. Some plants were recognized as medicinal, but the interviewees could not accurately recall their specific uses, suggesting that local knowledge associated with these plants is disappearing. With increasing deforestation, land-use change, and the influence of mainstream culture, this trend may accelerate further [60]. Meanwhile, the development of modern medicine and improved healthcare conditions have also affected the use of traditional medicine. As modern pharmacies and medical institutions become more common in both towns and rural areas, community residents are increasingly inclined to choose modern medical treatment for everyday illnesses.

### Conservation and sustainable use of medicinal plants and traditional knowledge

The traditional knowledge documented in this study should not remain merely at the level of literature preservation, but should be further transformed into community centered practices for conservation and sustainable use. Local herbalists and elders, as the main holders of Jinuo medicinal knowledge, should actively participate in relevant actions to ensure that conservation measures are consistent with the local cultural context and the practical needs of the community. In terms of medicinal plant resource conservation, efforts should be made to enhance community awareness of the value of medicinal plants and their associated traditional knowledge. The establishment of household medicinal plant gardens and community demonstration gardens could be encouraged, especially for species with high use frequency and those potentially affected by overharvesting. Such practices would not only help reduce dependence on wild populations, but also alleviate harvesting pressure. For example, some traditional Jinuo healers have already cultivated medicinal plants such as *Stephania epigaea* and *Gynostemma pentaphyllum* around their houses, indicating that small scale cultivation has a certain practical foundation in local communities. In the future, medicinal plant demonstration gardens could be established near villages, schools, or community activity centers, allowing them to serve simultaneously as sites for living germplasm conservation, community education, and intergenerational knowledge transmission.

In terms of knowledge transmission, training courses, plant identification activities, and knowledge exchange programs should be organized to encourage the participation of young people, women, and broader community members in learning traditional medicine, thereby gradually overcoming the limitations of transmission mainly within families and through male members. Meanwhile, local governments and relevant institutions could collaborate with herbalists to update and publish books or manuals related to Jinuo medicine, and use online platforms, local cultural websites, or digital media to increase public awareness of traditional medicine. Given that few new specialized works have appeared in the more than two decades since *Jinuo Medicine* was published in 2001, community participatory documentation, compilation, and publication of relevant materials would have practical significance for protecting local knowledge and supporting future research.

In addition, Jinuo communities should be encouraged to cooperate with medical colleges and research institutes in Xishuangbanna and Yunnan to explore the establishment of courses, training programs, or field teaching mechanisms related to Jinuo ethnomedicine. Such initiatives would enable more Jinuo community members to receive systematic training in traditional medicine, plant identification, sustainable harvesting, and basic health knowledge. Meanwhile, an electronic database for ethnic medicine could be established to document traditional Jinuo healers, medicinal plant species, and their resource status, thus supporting the long-term preservation of traditional knowledge and future research. Studies have shown that information systems play an important role in the protection and use of traditional knowledge [83]. Smith noted that such systems facilitate the documentation and retrieval of ethnobotanical information, help preserve traditional knowledge associated with medicinal plants, support the discovery of new plant-derived medicines, and contribute to sustainable healthcare practices [84, 85]. However, database construction should follow the principle of free, prior and informed consent, respect the rights of knowledge holders, protect sensitive prescriptions, and allow the community to determine the scope and mode of knowledge sharing. Through these specific pathways, the local knowledge documented in this study could be further transformed into practical resources for promoting biodiversity conservation, cultural inheritance, sustainable resource use, and community health development.

## Conclusion

This study is an ethnobotanical investigation of medicinal plants used by the Jinuo people, focusing on medicinal plants and their associated traditional knowledge in Jinuo communities. A total of 226 medicinal plant species were recorded, belonging to 197 genera and 90 families. These plants are used to treat 94 common ailments, mainly involving digestive system disorders, respiratory diseases, and traumatic injuries. Based on the analysis of RFC values, species such as *Plantago asiatica*, *Stephania epigaea*, *Tacca chantrieri*, *Gynostemma pentaphyllum*, and *Phyllanthus urinaria* were found to be of high local importance, representing key medicinal plants commonly used by traditional Jinuo healers and widely recognized by local villagers. In addition, this study documented 40 commonly used medicinal and edible plants, indicating that the Jinuo people have developed the concept of “food and medicine from the same source” through long-term practice. This not only reflects the practical value of traditional medical knowledge, but also shows its continuing role in daily health maintenance.

Although modern medicine has become widely available in the region, traditional Jinuo medicine still plays an indispensable role in primary healthcare and to some extent complements modern medical practice. However, the survey also showed that villagers familiar with medicinal plant knowledge are mainly concentrated in the 40–75 age group, indicating a clear aging trend among traditional knowledge holders. At the same time, the decline in the number of traditional Jinuo healers, together with the fact that many young people leave for education or migrant work, has placed traditional medical knowledge at continued risk of loss. Meanwhile, human disturbances such as the expansion of rubber and tea plantations, land-use change, and unregulated harvesting have also contributed to the decline of some wild medicinal plant resources, especially species whose roots or whole plants are used medicinally, as they are more vulnerable to harvesting pressure.

Therefore, the conservation of Jinuo medicinal plant resources and their associated traditional knowledge has become increasingly urgent. Future efforts should, with full respect for the wishes of local communities and the rights of traditional knowledge holders, systematically document information on traditional Jinuo healers, establish electronic databases for medicinal plants and traditional knowledge, and carry out urgent documentation through multiple forms such as audiovisual and written records. At the same time, training and guidance for community members, especially younger generations, should be strengthened to encourage their participation in traditional knowledge learning and medicinal plant cultivation. The development of home medicinal gardens and cultivated planting should also be promoted to reduce pressure on wild resources. Traditional Jinuo medicine is not only an important system for the use of biological resources, but also an important part of cultural heritage. Its effective conservation will help strengthen community cultural resilience and promote regional development, while also providing a reference for the screening and development of potential medicinal plants, as well as for the conservation and sustainable use of biological resources.

## Data Availability

All data generated or analyzed during this study were included in this article.

## Abbreviations

B: Bark
Bot: Both wild and cultivated
Cul: Cultivated
Fl: Flower
Fr: Fruit
H: Heartwood
JI: Jaccard similarity index
K: Kernel
L: Leaf
R: Root
RFC: Relative frequency of citation
Rh: Rhizome
Se: Seed
Sk: Skin of fruit
St: Stem
T: Tuber
W: Whole plant
Wil: Wild
